# Habitat Suitability of *Ixodes ricinus* Ticks Carrying Pathogens in North-East Italy

**DOI:** 10.3390/pathogens13100836

**Published:** 2024-09-27

**Authors:** Maartje Huitink, Myrna de Rooij, Fabrizio Montarsi, Maria Vittoria Salvati, Federica Obber, Graziana Da Rold, Sofia Sgubin, Elisa Mazzotta, Guido di Martino, Matteo Mazzucato, Cristiano Salata, Nicoletta Vonesch, Paola Tomao, Lapo Mughini-Gras

**Affiliations:** 1 Institute for Risk Assessment Sciences, Faculty of Veterinary Medicine, Utrecht University, Yalelaan 2, 3584 CL Utrecht, The Netherlands; m.huitink@uu.nl (M.H.); m.m.t.derooij@uu.nl (M.d.R.); 2 Istituto Zooprofilattico Sperimentale delle Venezie (IZSVe), Viale dell’Università 10, 35020 Legnaro, Italy; fmontarsi@izsvenezie.it (F.M.); fobber@izsvenezie.it (F.O.); gdarold@izsvenezie.it (G.D.R.); ssgubin@izsvenezie.it (S.S.); emazzotta@izsvenezie.it (E.M.); gdimartino@izsvenezie.it (G.d.M.); mmazzucato@izsvenezie.it (M.M.); 3 Department of Molecular Medicine, University of Padua, Via Gabelli, 63, 35121 Padua, Italy; mariavittoria.salvati@phd.unipd.it (M.V.S.); cristiano.salata@unipd.it (C.S.); 4 Department of Occupational and Environmental Medicine, Epidemiology and Hygiene, Italian Workers’ Compensation Authority (INAIL), Via Fontana Candida 1, 00078 Monte Porzio Catone, Italy; n.vonesch@inail.it (N.V.); p.tomao@inail.it (P.T.); 5 Centre for Infectious Disease Control, National Institute for Public Health and the Environment (RIVM), Antonie van Leeuwenhoeklaan 9, 3721 MA Bilthoven, The Netherlands

**Keywords:** *Ixodes ricinus*, Maxent, ecological niche modelling, tick-borne diseases, Italy

## Abstract

*Ixodes ricinus* ticks are ubiquitous in Europe, including in North-East Italy. These ticks are important vectors of several zoonotic pathogens of public health relevance. In this study, the habitat suitability range of *I. ricinus* ticks infected with zoonotic pathogens was predicted in North-East Italy, and relevant spatial predictors were identified. In 2015–2021, ticks were collected at 26 sampling sites in the study area. The collected ticks were screened for the presence of pathogens using PCR assays. For *Borrelia*, *Rickettsia* and *Anaplasma/Ehrlichia* species, data allowed for ecological niche modelling using Maxent. Environmental determinants potentially related to tick habitat suitability were used as model inputs. Predicted suitable habitat distributions revealed hotspots of the probability of pathogen presence in *I. ricinus* ticks mainly in the central and upper parts of the study area. Key environmental predictors were temperature, rainfall and altitude, and vegetation index for specific pathogens (*Rickettsia* and *Anaplasma*/*Ehrlichia* species). Increased risk of exposure to tick-borne pathogens upon tick bites in the predicted hotspot areas can, therefore, be expected. This provides useful information for public health risk managers in this and other similar regions.

## 1. Introduction

*Ixodes ricinus* (Linnaeus, 1758; Parasitiformes: Ixodida) is widespread in Europe, including in North-East Italy, where this species is the most common tick vector for numerous pathogens [1,2]. The pathogen prevalence among individually screened *I. ricinus* ticks in North-East Italy in 2011–2017 was about 40% [3]. Research by Rezza et al. estimated an annual tick bite incidence of 0.10% among humans in 2000–2013 [4]. Pathogens transmitted by *I. ricinus* ticks are, for example, *Borrelia*, *Rickettsia*, *Anaplasma* and *Ehrlichia* species, as well as viruses, such as tick-borne encephalitis virus [4]. 

The prevalence and distribution of *I. ricinus* ticks and associated pathogens are influenced by both host-related and environmental factors [5,6,7], which show a complex and dynamic interplay [8]. Due to small-scale geographical variation in these factors, research focused on specific areas enables prediction of the distribution of ticks and tick-borne pathogens [8]. Habitat suitability modelling is a valid approach to predicting species distribution and can be applied when detailed geolocated data on environmental factors and tick/pathogen abundance data are available [9,10]. Earlier research showed that temperature and humidity are significant spatial predictors of *I. ricinus* abundance, with an association resembling an inverted U-shape: high temperature and humidity favor tick abundance [6,11,12]. Other significant predictors are altitude, rainfall and the Normalized Difference Vegetation Index (NDVI), with a negative association for altitude and a positive association for rainfall and NDVI [5,13,14]. Forests are preferred over meadows and other open areas by *I. ricinus*; thus, land cover is also a significant predictor [12,13]. North-East Italy provides favorable environmental conditions for the presence of *I. ricinus,* as well as for the survival and maintenance of *I. ricinus*-carried pathogens [15,16]. Risk characterization in this area is important due to the upsurge in human cases of tick-borne disease and its geographical position, being at the crossroads between the Mediterranean Sea, Western Europe and the Balkans [16,17]. 

The aim of this study was to identify geographical hotspots in terms of the probability of exposure to tick-borne pathogens upon a tick bite in North-East Italy. Moreover, this study aimed at exploring environmental predictors of the presence of *I. ricinus* ticks infected with pathogens. This aim was pursued by estimating (i) the habitat suitability range for *I. ricinus*-borne pathogens in the area and (ii) the relevance of spatial predictors regarding the habitat suitability range. 

## 2. Materials and Methods

### 2.1. Study Design and Data Collection

Samples were collected in North-East Italy. This area is divided into three administrative regions: Veneto, Trentino-Alto Adige and Friuli Venezia Gulia. The area is characterized by pre-Alpine and Alpine areas, a sub-continental climate, a relatively high humidity of around 85% and moderate precipitation of around 1000 millimeters per year [15,16]. 

**Figure 1:**
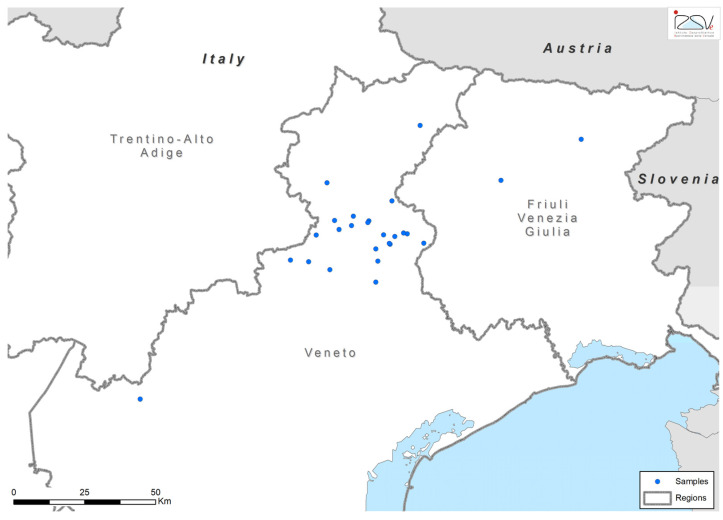
Geographical distribution of the 26 sampling sites in North-East Italy.

From 2015 to 2021, data were collected at 26 different sampling sites distributed throughout Veneto and Friuli Venezia Giulia (specifically in the provinces of Belluno, Pordenone, Treviso, Udine and Verona), as part of entomological surveillance activities conducted by the Instituto Zooprofilattico Sperimentale delle Venezie (IZSVe). Figure 1 shows the distribution of the sampling sites. Geographical data, such as latitude and longitude, were recorded during the sampling at each site and are specified in Supplementary Table S1. The annual mean temperature at the sampling sites was 9.78 °C, mean annual precipitation was 41.65 mm and mean altitude was 846.47 m above sea level. Mean temperature was 19.64 °C during the warmest quarter and −0.28 °C during the coldest quarter. The 26 sites were selected based on tick occurrence data reported after previous samplings in the same study area and, therefore, prior knowledge of the potential distribution of *I. ricinus* in the region [1,15,16]. From 2015–2021, sites were sampled from March to November, with a frequency varying from one to 15 times during the study period. Ticks were collected by standard dragging a 1 m^2^ white flannel cloth over the ground and vegetation along 50 m, stopping for collection every 2.5 m to prevent detachment of the ticks. The collected ticks were identified morphologically using the keys for Italian tick species [18,19] pooled according to the stage (single adult male or female and maximum of 10 specimens of larvae or nymphs), and stored at −80 °C until molecular analysis for pathogen identification. 

### 2.2. Molecular Detection of Tick-Borne Pathogens

The ticks were screened by molecular techniques for the detection of different pathogens: Tick Borne Encephalitis (TBE) virus, *Borrelia, Rickettsia, Anaplasma, Ehrlichia* and *Babesia* species. In addition, screening for Crimean Congo Haemorrhagic Fever (CCHF) virus was performed to search for this pathogen in North-Italy. Samples were homogenized in 600 μL of Phosphate Saline Buffer (PBS) adding a 5 mm bead (Qiagen, Venlo, The Netherlands) and using the TissueLyser II (Qiagen) at a room temperature of 24 °C. Nucleic acids (DNA and RNA) were extracted using the Maxwell^®^ CSC Viral Total Nucleic Acid Purification Kit designed for the Maxwell^®^ RSC48 Instrument (Promega, Madison, Wisconsin, USA), according to the manufacturer’s instructions, and then kept frozen at −80 °C. Target genes and primers used for testing, the size of amplicons and the references are reported in Supplementary Table S2. As extraction control, a real-time PCR targeting the 18S rRNA of *I. ricinus* was adopted [20]. 

A real-time PCR was used. Briefly, for DNA pathogens, nucleic acids were detected using the SYBR™ Green PCR Master Mix (Applied Biosystems™, Waltham, Massachusetts, USA) in a QuantStudio™ 3 Real-Time PCR System (Applied Biosystems™) with a thermal cycling profile consisting of an initial denaturation/DNA polymerase activation at 95 °C for 10 min, followed by 40 cycles of denaturation at 95 °C for 10 sec and annealing/extension at 60 °C for 1 min. The specificity of fluorescence signal was evaluated with melting curve analyses, according to the manufacturer’s instructions. Since the PCR used was not able to discern between *Anaplasma* and *Ehrlichia* species, these two genera are hereafter reported combined as *Anaplasma/Ehrlichia*. Positive samples for *Borrelia* spp., *Anaplasma/Ehrlichia* spp. and *Rickettsia* spp. were subject to further nested PCR analyses. Nested PCR analyses were performed using the TransTaq^®^ DNA Polymerase High Fidelity (TransGen Biotech, Beijing, China), following the published information and also further described in Supplementary Table S2. For TBE virus and CCHF virus, a reverse transcription was carried out using random primers (Invitrogen™, Carlsbad, California, USA) and HiScript III RT SuperMix (+gDNA wiper) (Vazyme, Nanjing, China) following the manufacturer’s instructions. Thus, cDNA was subject to real-time PCR amplification as above reported. Finally, *Babesia* spp. was detected by an end-point PCR and sequencing.

### 2.3. Environmental Data 

Environmental predictors consisted of meteorological and elevation data, as well as data on vegetation and land cover, at a spatial resolution of 1 km^2^. Bioclimatic variables were downloaded from the WorldClim database [21]. The 19 biovars of WorldClim were calculated for the years 2015–2021, based on North-East Italy’s meteorological data for the years 2015–2020, relying on rainfall data from the meteorological stations in the study area and the Land Surface Temperature (LST) from Moderate Resolution Imaging Spectroradiometer (MODIS) satellite images (continuity in data was assumed towards the year 2021). The digital terrain model (DTM), a representation of elevation above sea level in meters, was obtained from the 2011 version of DTM, from the satellite constellation named ‘Satellite Pour l’Observation de la Terre’ (SPOT), with the original spatial resolution of 25 m resampled to 100 m. NDVI, used as a proxy for density and health status of vegetation (expressed as a range from −1 to 1), was averaged over seasons: spring (March–May), summer (June–August), autumn (September–November) and winter (December–February), across the six study years. The original satellite images were obtained from Copernicus’s Sentinel-2 satellites, with an original spatial resolution of 10 m. The seasonal average values were resampled to 1 km. The Corine Land Cover (CLC) level 2 (2018 version) was chosen to represent the land cover use and was obtained from the Copernicus portal (https://land.copernicus.eu/pan-european/corine-land-cover, accessed on 1 October 2021) [22]. All data described above were derived from elaborations of the EVE system within IZSVe [23]. The spatial predictors used for modelling are listed in Table 1. Descriptive statistics of these data at the sampling sites were obtained through a spatial join in ArcGIS. 

### 2.4. Habitat Suitability Modelling 

Ecological niche modelling was performed using Maxent, version 3.4.4 [24]. Models were generated for the region of North-East Italy. Four habitat suitability models were created for pathogen presence in *I. ricinus* ticks: (i) any pathogen detected (all pathogen species together), (ii) *Borrelia* spp., (iii) *Rickettsia* spp. and (iv) *Anaplasma*/*Ehrlichia* spp. detected. For *Babesia* spp., the number of positive samples was too limited for modelling purposes (n = 30, see below).

#### 2.4.1. Probability of Pathogen Presence in Ticks

To model the probability of the presence of any of the pathogens altogether, and of each pathogen species separately, in the ticks, data on pathogen detection in the ticks collected at the sampling sites combined with the corresponding spatial predictors were used as input for Maxent. One model for all pathogens together and one model per pathogen were built. Default settings were used, with a test percentage of 25 and the option to perform a jackknife test. The output format was set at “Cloglog”—the default setting—to estimate the probability of the presence of pathogens in the ticks in the area.

#### 2.4.2. Model Assessment 

Following standard procedures, the models produced by Maxent were assessed by evaluating omission rate and area under the curve (AUC) from the receiver operating characteristic (ROC) curve [24,25]. To identify the most important spatial predictors of habitat suitability, the percent contribution and permutation importance of the spatial determinants, ranging from 0–100%, were assessed and combined with information regarding gains and AUC per variable from the jackknife test [24,25]. In addition to the habitat suitability modelling using Maxent, four generalized additive models (GAMs) were built using R, R studio and the mgcv, mass, corrplot, raster and ggplot2 packages [26,27]. These additional analyses were performed for comparison purposes only, to produce habitat suitability results based on more classical statistical modelling approaches that do not rely on machine-learning methods (i.e., Maxent) (Supplementary Methods). 

## 3. Results

### 3.1. Tick and Tick-Borne Pathogen Detection 

Ticks were found at all sampling sites at every sampling round. In total, 3133 ticks belonging to *I. ricinus* were collected during 98 samplings moments: larvae (n = 493), nymphs (n = 2402) and adults (n = 125 males and n = 113 females). No other tick species were collected. Different life stages and combinations thereof, i.e., only larvae, only nymphs, only adults, larvae and nymphs, larvae and adults, nymphs and adults, and all life stages, were found and tested for pathogens in, respectively, 1, 49, 7, 2, 1, 26 and 12 sampling moments. The minimum and maximum number of ticks collected per sampling moment was 1 and 256, respectively. A total of 346 pooled samples (all stages) were tested for pathogen detection (32, 219 and 95 pools of larvae, nymphs and adults, respectively). Overall, one or more pathogens were detected in 64% of the screened pools; 38% were positive for *Borrelia* spp., 35% for *Rickettsia* spp., 26% for *Anaplasma*/*Ehrlichia* spp. and 17% for *Babesia* spp. However, *Babesia* spp. could only be tested in the samples collected in 2020–2021 (24 sampling moments and 176 pooled samples). All samples were negative for TBE and CCHF viruses. Pathogen presence was detected predominantly in nymphs, to a lesser extent in adult ticks and to an even lesser extent in larvae. More detailed descriptive statistics regarding ticks (Supplementary Table S3), the presence of pathogens per tick life stage (Supplementary Table S4), the presence of pathogens at each sampling site (Supplementary Table S5), spatial predictors in the studied areas (Supplementary Table S6) and spatial predictors at the sampling sites (Supplementary Table S7) are provided in the Supplementary Materials. 

### 3.2. Model Results for Probability of Pathogen Presence

**Figure 2:**
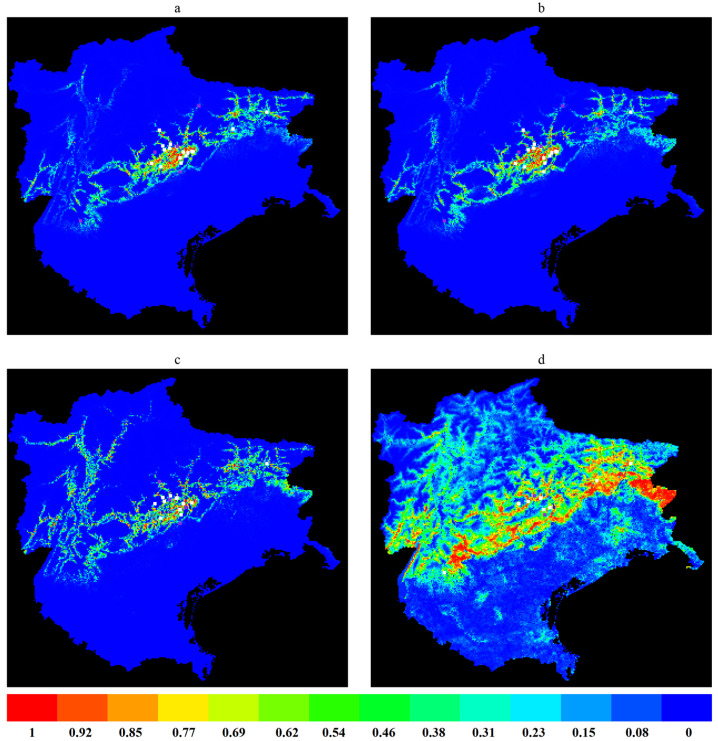
Habitat suitability maps from Maxent for *I. ricinus* ticks carrying pathogens in North-East Italy. (**a**) Presence of any pathogen carrying tick. (**b**) Presence of *Borrelia*-carrying ticks. (**c**) Presence of *Rickettsia*-carrying ticks. (**d**) Presence of *Anaplasma/Ehrlichia*-carrying ticks. Warmer colors indicate a better habitat suitability. Colors correspond to the probability of presence; red to 1.00, orange to 0.85–0.92, yellow to 0.77, green to 0.46–0.69 and blue to 0.00–0.38. White dots show the presence locations used for training and violet dots show the test locations of the accompanying model.

Figure 2 shows the habitat suitability distribution map produced by Maxent for the probability of presence of *I. ricinus* ticks infected with any of the tested pathogens, as well as the maps specific for *Borrelia* spp., *Rickettsia* spp. and *Anaplasma/Ehrlichia* spp. separately. The main model results are presented here below, while further details on all model outputs are provided in the Supplementary Materials.

For the probability of any pathogen presence in ticks, the spatial predictors used for habitat suitability modelling that had the biggest influence were DTM (30.3 percent contribution [PC], 50.2 permutation importance [PI] and 0.90 AUC), Bio 13 (29.2 PC, 2.8 PI and 0.70 AUC) and Bio 11 (5.7 PC, 39.6 PI and 0.87 AUC). These spatial predictors were also the most influencing ones for the probability of *Borrelia* spp. presence: Bio 13 (34.2 PC, 6.2 PI and 0.87 AUC), DTM (30.3 PC, 52.7 PI and 0.87 AUC) and Bio 11 (0.7 PC, 25.1 PI. 0.90 AUC). NDVI in summertime was the most influencing variable for both *Rickettsia* spp. (49.8 PC, 27.6 PI and 0.86 AUC) and *Anaplasma*/*Ehrlichia* spp. (32.0 PC, 13.6 PI and 0.90 AUC). This was followed by Bio 11 and DTM for *Rickettsia* spp. (27.6 PC, 38.3 PI and 0.88 AUC, and 10.5 PC, 25.9 PI and 0.89 AUC, respectively) and Bio 7 for *Anaplasma*/*Ehrlichia* spp. (9.2 PC, 42.9 PI and 0.65 AUC).

The performance of the models for any pathogen presence, for *Borrelia* spp. and *Rickettsia* spp., showed an omission that matched the predicted omission, which was better than random and useful in predicting the probability of presence. The AUC of the training data versus testing data was 0.99 versus 0.91 (any pathogen), 0.99 versus 0.89 (*Borrelia* spp.), and 0.98 versus 0.92 (*Rickettsia* spp.). Conversely, the performance of the model for *Anaplasma*/*Ehrlichia* showed an omission that did not match the predicted omission well; thus, more data would be needed to predict the probability of presence of *Anaplasma*/*Ehrlichia*-infected ticks more precisely (AUC training data versus testing data was 0.95 versus 0.97). 

DTM was the variable with the highest gain when used in isolation and also the variable that decreased the gain the most when omitted in the models for any pathogen presence and for *Borrelia* spp. For *Rickettsia* spp., Bio 11 was the variable with the highest gain when used in isolation and NDVI in summertime was the one that decreased the gain the most when omitted. For *Anaplasma*/*Ehrlichia* spp., NDVI in summertime and Bio 7 were the variables with the highest gain when used in isolation and that decreased the gain the most when omitted, respectively. 

Additional analyses performed by GAM to enable comparisons to Maxent models showed results to be mostly comparable (see Supplementary Results). GAM results indicated NDVI in springtime as an additional spatial determinant of the probability of the presence of infected ticks and Bio 18 as an additional spatial determinant for *Anaplasma*/*Ehrlichia* spp.

## 4. Discussion

This study aimed to provide insights into the habitat suitability range for *I. ricinus* ticks infected with public health-relevant pathogens in North-East Italy, including an assessment of the spatial predictors of such range. The overall pathogen prevalence (*Borrelia*, *Rickettsia* and *Anaplasma*/*Ehrlichia* spp. combined) was 64%, as determined in the 346 pooled samples of ticks analyzed here. This is higher than previously reported (40%) in individual tick samples from the same study area [3]. In this study, TBE en CHHF viruses were not detected, although TBE has been detected in the study area before [15] and *I. ricinus* is described as a potential vector for CCHF virus [28]. 

Pathogen presence was detected predominantly in nymphs, and to a lesser extent in adult ticks and larvae. Therefore, the models mainly predicted the pathogen presence in nymphs. Due to their relatively small size, large numbers and activity, nymphs pose a higher public health risk compared to adults and larvae [29]. The habitat suitability maps showed the highest probabilities of the presence of *Anaplasma*/*Ehrlichia*-infected ticks to be located mainly in the central and upper parts of the study area. The highest probabilities of the presence of ticks infected with any pathogen, as well as, specifically *Borrelia*- and *Rickettsia*-infected ticks, were mostly located in the center of the study area. This is essential information to inform public health interventions. 

Important spatial predictors of presence of *I. ricinus* ticks carrying pathogens were temperature, rainfall, DTM and NDVI. Tick development is mainly influenced by temperature and humidity [30]. North-East Italy has a sub-continental climate. Temperature is relatively high, especially in summer [15,16]. Humidity, affected by water vapor—which is influenced by rainfall and temperature—is relatively high as well [15,31]. It is, therefore, plausible that these factors influence tick development in the study area positively and, consequently, a relatively high number of ticks and associated pathogens can be expected [5,30]. The mean altitude in the study area, as calculated based on DTM, is 846.47 m above sea level. In Italy, ticks only become far less abundant at a higher altitude of 1300 m above sea level [8]. This higher altitude coincides with lower temperature and open habitats, with less favorable microclimatic conditions and host availability [8,14]. The mean NDVI in the study area is 0.41, meaning that vegetation is moderately dense and healthy [32]. Dense and healthy vegetation can provide cover and litter depth, which creates favorable microclimatic conditions for ticks and pathogen survival [14]. Furthermore, vegetation is required for questing, thereby making completion of the tick life-cycle and spread of pathogens possible [30]. 

Land cover was not identified as a significant spatial predictor in this study, but was described as possibly relevant to the occurrence of *I. ricinus* ticks and associated pathogens in previous research in North-East Italy [12,13]. Land cover influences the distribution of host species and provides microclimatic conditions favorable to tick survival. However, compared to, e.g., temperature and humidity, it does not play an important role in influencing tick behavior like host-seeking activity, which, in turn, is important for key vital rates and pathogen occurrence [33]. Given the favorable temperature, humidity and NDVI in the study area, it can be assumed that these variables were stronger predictors than land cover.

The results substantiate current knowledge regarding the occurrence of *I. ricinus* and associated pathogens, specifically *Borrelia* spp., *Rickettsia* spp. and *Anaplasma*/*Ehrlichia* spp., in North-East Italy. Given the extent of the study area, the number and geographical distribution of sampling sites in this study can be considered limited. The sampling sites were chosen based on the accessibility and consistency of sampling with prior knowledge of the potential distribution of *I. ricinus* in the region, but some sites were sampled more often than others. Therefore, we cannot exclude that sampling bias might have occurred. Consequently, generalizations of study findings, including predictions based on the models, especially regarding areas not covered by the sampling sites, should be made with caution. However, the findings of field studies in North-East Italy [34,35] conducted in areas not covered by our sampling sites were in agreement with our model predictions [34,35]. While the spatial representativeness of sampling is important, especially in cases where the distribution is completely unknown, the results of previous surveys indicate that sampling covered the true distribution of the species under study. In general, Maxent is known to predict conservatively around sampling areas; therefore, the findings should be considered in light of the potential underestimation of the extent of the hotspot areas [36]. The predictive capabilities of the model are deemed limited outside the environmental ranges that define areas different from those in which the information has been obtained (i.e., the sampling sites). However, considering the region in our study, such drastic environmental heterogeneities are not present. Lastly, this study’s findings contribute to assessing public health relevance and infectious disease risks, albeit only partly. On the one hand, the pathogens studied are not only transmitted by *I. ricinus* but by other tick species as well. On the other hand, the pathogenicity can vary between pathogen species of the same genera, which we could not differentiate with the PCR assays used. 

The risk for people bitten by *I. ricinus* ticks being exposed to *I. ricinus*-borne pathogens can be considered to be relatively higher in the predicted hotspot areas. It is important to highlight that some occupational categories are at increased risk, particularly outdoor workers, including forestry and agricultural workers, farmers and landscape and wildlife management workers [3]. Infection with any of the pathogens can result in, often flu-like, clinical symptoms in humans, but also in gastro-intestinal problems, respiratory distress and neurological symptoms [37,38,39,40]. Targeted awareness campaigns amongst local communities, as well as preparedness in terms of both preventive and responsive measures, could mitigate the health risks resulting from exposure to tick-borne pathogens. Moreover, the identification of infected ticks in a focused geographic area could assist occupational physicians, in accordance with the regulations on the health and safety of workers exposed to biological agents, in carrying out appropriate health surveillance of workers at risk of exposure [41]. Future research could focus on risk mapping investigating spatial patterns and potential changes over time, determining the tick bite incidence in North-East Italy and monitoring prevalence of infection with any of the pathogens after a tick bite. This would then promote localizing areas with an increased disease risk, thus allowing for the application of more targeted and effective protection measures. 

The disease burden due to tick-borne pathogens is on the rise [40]. Climate change is suggested to be of influence. Since 1981, the mean annual temperature on Earth has risen by 0.18 degrees Celsius per decade [42]. The majority of ticks prefer relatively higher temperatures and warmer climates [30]. Therefore, a climate change-driven rise in the global mean annual temperature is likely to lead to an increase in the occurrence of ticks and pathogens already found in the study region and beyond, but also to an increase in emerging pathogens, which could have negative implications for both animal and human health. One of these emerging pathogens is CCHF virus. Infection in humans can evolve from flu-like illnesses to serious hemorrhagic syndromes, with a fatality rate of up to 40% [17]. While a current occurrence of CCHF virus in North-East Italy seems unlikely, the virus is present in the neighboring Balkans [17]. Since North-East Italy forms the doorstep from the Balkans to the rest of Italy, a rise in the mean annual temperature may very well lead to the introduction of CCHF virus in Italy, which warrants vigilance and continuing surveillance efforts. Besides *I. ricinus*, also other tick species present in Italy may then be relevant in efficiently spreading the CCHF virus [17]. CCHF virus has been detected in at least 31 tick species [28]. In particular, *I. ricinus* infected by CCHF virus has been reported in endemic regions, such as Kosovo and Bulgaria. A recent systematic review suggests that *I. ricinus* can be a potential vector for CCHF virus [28]. Considering the distribution of *I. ricinus* in the study area and the close proximity to endemic regions, we cannot exclude a contribution of *I. ricinus* in CCHF virus diffusion in the area. 

## 5. Conclusions

In conclusion, one or more pathogens were detected in 64% of screened pools of *I. ricinus* ticks, with *Borrelia*, *Rickettsia* and *Anaplasma*/*Ehrlichia* spp. being the most prevalent pathogens found, whereas no TBE or CCHF viruses were detected. Ecological niche modelling using Maxent showed that the study area provides widespread suitable habitats for *I. ricinus* ticks infected with *Borrelia* spp., *Rickettsia* spp. and *Anaplasma*/*Ehrlichia* spp. Several key environmental predictors of the presence of these pathogens in ticks were also identified, with temperature, rainfall and altitude playing a prominent role, alongside vegetation index for *Rickettsia* and *Anaplasma*/*Ehrlichia* spp., specifically. Hotspots of the probability of pathogen presence—and, therefore, risk of exposure to these pathogens upon tick bite—were also identified, particularly in the central and upper parts of the study area. These hotspots allow for targeted awareness campaigns amongst local communities to mitigate the health risks resulting from exposure to these tick-borne pathogens. 

## Figures and Tables

**Table 1:** Spatial predictors used in generating habitat suitability models.

Spatial Predictors	Code	Source
Annual Mean Temperature	Bio 1	WorldClim *^2^
Mean Diurnal Range	Bio 2	WorldClim
Isothermality	Bio 3	WorldClim
Temperature Seasonality	Bio 4	WorldClim
Maximum Temperature of Warmest Month	Bio 5	WorldClim
Minimum Temperature of Coldest Month	Bio 6	WorldClim
Temperature Annual Range	Bio 7	WorldClim
Mean Temperature of Wettest Quarter	Bio 8	WorldClim
Mean Temperature of Driest Quarter	Bio 9	WorldClim
Mean Temperature of Warmest Quarter	Bio 10	WorldClim
Mean Temperature of Coldest Quarter	Bio 11	WorldClim
Annual Precipitation	Bio 12	WorldClim
Precipitation of Wettest Month	Bio 13	WorldClim
Precipitation of Driest Month	Bio 14	WorldClim
Precipitation Seasonality	Bio 15	WorldClim
Precipitation of Wettest Quarter	Bio 16	WorldClim
Precipitation of Driest Quarter	Bio 17	WorldClim
Precipitation of Warmest Quarter	Bio 18	WorldClim
Precipitation of Coldest Quarter	Bio 19	WorldClim
Digital Terrain Model	DTM	SPOT *^3^
Normalized Difference Vegetation Index	NDVI	COPERNICUS *^4^
Corine Land Cover, level 2 *^1^	CLC	COPERNICUS

*^1^ Categorical variable. *^2^ WorldClim is a database of global weather and climate data. The data are provided to be used for mapping and spatial modelling in research and related activities (https://www.worldclim.org/, accessed on 1 October 2021) [21]. Elaborations were performed within the EVE system, staged in the IZSVe infrastructure [23]. *^3^ SPOT is a satellite constellation named ‘Satellite Pour l’Observation de la Terre’. Elaborations were performed within the EVE system, staged in the IZSVe infrastructure [23]. *^4^ COPERNICUS provides a portal from which data on, i.e., land cover use can be obtained (https://land.copernicus.eu/pan-european/corine-land-cover, accessed on 1 October 2021) [22]. Elaborations were performed within the EVE system, staged in the IZSVe infrastructure [23].

## Data Availability

The original data presented in the study are openly available in the public repositories and previous publications mentioned in the manuscript.

## Supplementary Materials

The following supporting information can be downloaded at: https://www.mdpi.com/article/10.3390/pathogens13100836/s1, Supplementary Methods; Supplementary Results; Table S1: Latitude and longitude in World Geodetic System 1984 (WGS84) of each sampling site; Table S2: PCR systems used for tick-borne pathogen detection; Table S3: Descriptive statistics of tick data; Table S4: Presence of pathogens per tick life stage; Table S5: Presence of pathogens at each sampling site; Table S6: Descriptive statistics of spatial determinants in North-East Italy; Table S7: Descriptive statistics of spatial determinants at the sampling sites; Table S8: Results of univariable regression analysis, Pathogens; Table S9: Results of univariable regression analysis, Borrelia spp.; Table S10: Results of univariable regression analysis, *Rickettsia* spp.; Table S11: Results of univariable regression analysis, *Ehrlichia* spp.; Figure S1: Correlation matrix spatial determinants; Figure S2: Habitat suitability maps for North-East Italy from GAM; Supplementary details on Maxent modelling; Supplementary references. References [20,26,27,43,44,45,46,47,48,49,50,51,52,53,54,55] are cited in the Supplementary Materials.
