# Supplementary material for: Habitat Suitability of Ixodes ricinus Ticks Carrying Pathogens in North-East Italy
Source: Pathogens. 2024 Sep 27;13(10):836. doi: 10.3390/pathogens13100836 (PMC11510671; doi:10.3390/pathogens13100836)
Supplement: Supplementary file 1 [file pathogens-13-00836-s001.zip › pathogens-3201679-supplementary.pdf]

SUPPLEMENTARY MATERIAL

Article

Habitat Suitability of *Ixodes ricinus* Ticks Carrying Pathogens in North-East Italy

Maartje Huitink [1], Myrna de Rooij [1], Fabrizio Montarsi [2], Maria Vittoria Salvati [3], Federica Obber [2], Graziana Da Rold [2], Sofia Sgubin [2], Elisa Mazzotta [2], Guido di Martino [2], Matteo Mazzucato [2], Cristiano Salata [3], Nicoletta Vonesch [4], Paola Tomao [4] and Lapo Mughini-Gras [1,5,*]

[1] Institute for Risk Assessment Sciences, Faculty of Veterinary Medicine, Utrecht University, Yalelaan 2, 3584 CL Utrecht, The Netherlands
[2] Istituto Zooprofilattico Sperimentale delle Venezie (IZSVe), Viale dell'Università 10, 35020 Legnaro, Italy
[3] Department of Molecular Medicine, University of Padua, Via Gabelli, 63, 35121 Padua, Italy
[4] Department of Occupational and Environmental Medicine, Epidemiology and Hygiene, Italian Workers' Compensation Authority (INAIL), Via Fontana Candida 1, 00078 Monte Porzio Catone, Italy
[5] Centre for Infectious Disease Control, National Institute for Public Health and the Environment (RIVM), Antonie van Leeuwenhoeklaan 9, 3721 MA Bilthoven, The Netherlands
* Correspondence: l.mughinigras@uu.nl or lapo.mughini.gras@rivm.nl

TABLE OF CONTENTS

SUPPLEMENTARY METHODS...3
SUPPLEMENTARY RESULTS...6
SUPPLEMENTARY TABLES..8
SUPPLEMENTARY FIGURES..32
SUPPLEMENTARY DETAILS ON MAXENT MODELLING..35
SUPPLEMENTARY REFERENCES...47

SUPPLEMENTARY METHODS

Habitat suitability modelling using generalized additive models

In addition to the habitat suitability modelling using Maxent, four generalized additive models (GAMs) were built using R, R studio and the mgcv, mass, corrplot, raster and ggplot2 packages [26,27,43–47]. This was done for comparison purposes to produce habitat suitability results based on more classical statistical modelling approaches that do not rely on machine-learning methods (i.e. Maxent). First, descriptive statistics were calculated and correlations between the independent variables were specified, after which the GAMs were built and sensitivity analyses were performed. Given the models from Maxent and the GAMs being build up in different ways, regarding each different dependent variable – all pathogens together and separately (*Borrelia, Rickettsia* and *Anaplasma*/*Ehrlichia*) – the models from Maxent and the GAMS are considered complementary to each other.

Correlations

In the GAM analysis, to identify multicollinearity issues among spatial determinants, the correlations between all these variables was investigated in a correlation matrix (Supplementary Figure S1). If the correlation between two variables was higher than (-)0.9, a choice was made between the two variables based on substantive grounds of relevance for the analysis. This led to the exclusion of Bio 5, Bio 10, Bio 11, Bio 13, Bio 16, Bio 17 and Bio 19 from the analyses, unless these variables had already proven to be important in the Maxent models. Therefore, Bio 11 was included in the analyses for the pathogens.

Modelling

Initially, for every dependent variable – pathogens, *Borrelia* species, *Rickettsia* species and *Anaplasma*/*Ehrlichia* species – a GAM was chosen over a generalized linear model (GLM), because data from the literature made it plausible for a relation between the

dependent and the independent variables to be represented by a non-linear curve, thus showing a potential optimal range for the spatial determinants in connection to the occurrence of the dependent variable. Regarding pathogens, *Borrelia*, *Rickettsia* and *Anaplasma*/*Ehrlichia* species, four GAMs were produced, which, considering the outcome of the dependent variables, were assigned to the family 'Binomial'.

Modelling started with a univariable regression analysis; the results can be found in the supplementary material (Supplementary Table S8 – Table S11). A p-value forming the cut-off value was decided upon, resulting in a maximum of ten independent variables for the next modelling step. This led to a cut-off value of $p < 0.10$ for pathogens in general, $p < 0.15$ for *Borrelia* species, $p <0.05$ for *Rickettsia* species and $p < 0.15$ for *Anaplasma*/*Ehrlichia* species. The model fit determined the order of importance of the remaining independent variables for the next modelling step. In order to generate final models for the dependent variables, forward and backward selection based on the Akaike information criterion (AIC) was performed on the remaining independent variables. Number of ticks was used as an offset variable when generating the final models for pathogens, *Borrelia*, *Rickettsia* and *Anaplasma*/*Ehrlichia* species, since the number of ticks collected varied per sampling moment and considering that higher numbers of ticks would lead to a higher probability of finding pathogens. Of the final models generated per dependent variable, by both forward and backward selection, the model with the lowest AIC was taken as the most important final model. Then, based on these final models, the resulting habitat suitability maps were created.

Sensitivity analyses

Model assumptions related to multicollinearity, potential influential outliers and linearity, independence and homoscedasticity of the residuals were checked for all final models. When the variance inflation factors (VIFs) were higher than 4.00 and therefore indicated possible problematic multicollinearity, the variable with the high(est) VIF was

removed from the model and the AIC and VIFs of the new model were checked, in order to see if the model improved. When Cook's distance was greater than 1.00 and thus indicative of a potential influential outlier, the observation was removed from the dataset and the model rerun and results compared. When the final model did not change, it was seen as reliable. Furthermore, it was checked for all final models, whether smooth terms or linear terms had the best fit to the data, by means of an ANOVA.

SUPPLEMENTARY RESULTS

GAM results for presence of pathogen-carrying ticks

The spatial determinants that were most important in the GAM for pathogen-carrying ticks were Bio 4 and NDVI in springtime. Both forward and backward selection led to the same final model, with linear terms (p-value ANOVA was 0.17) and an AIC of 92.31. Bio 4 and NDVI in springtime both showed a positive association with pathogen-carrying ticks. Supplementary Figure S2 shows the habitat suitability map based on the GAM. Model assumptions were met and there were no indications for potential influential outliers (VIF Bio 4 and NDVI in spring: 1.01, Cook's distance of 0.26).

GAM results for presence of *Borrelia*-carrying ticks

The spatial determinants that were most important in the GAM for *Borrelia*-carrying ticks were Bio 1 and Bio 7. Forward selection led to the final model, with smooth terms (p-value ANOVA was 0.00023) and an AIC of 123.87. Given the smooth terms, the GAM did not show indications regarding the association between the spatial determinants and *Borrelia*-carrying ticks. Supplementary Figure S2 shows the habitat suitability map based on the GAM. Initially, Bio 2 was included in the final model, but this spatial determinant was removed due to a VIF of 4.72. Furthermore, given a Cook's distance of 12.96, a potential influential outlier in the form of data from site 5038, was removed. After this, the model was rerun. Model assumptions were then met and there were no more indications for potential influential outliers (VIF Bio 1 and Bio 7: 1.39, Cook's distance of 0.30).

GAM results for presence of *Rickettsia*-carrying ticks

The spatial determinants that were most important in the GAM for *Rickettsia*-carrying ticks were Bio 4 and Bio 8. Forward selection led to the final model, with linear terms (p-

value ANOVA was 0.137) and an AIC of 84.54. Temperature seasonality and mean temperature of wettest quarter both showed a positive association with the presence of *Rickettsia*-carrying ticks. Supplementary Figure S2 shows the habitat suitability map based on the GAM. Model assumptions were met and there were no indications for potential influential outliers (VIF Biovars 4 and Biovars 8: 1.19, Cook's distance of 0.25).

GAM results for presence of *Anaplasma/Ehrlichia*-carrying ticks

The spatial determinants that were most important in the GAM for *Anaplasma/Ehrlichia* -carrying ticks were Bio 18 and Bio 7. Backward selection led to the final model, with smooth terms (p-value ANOVA was 0.0029) and an AIC of 80.84. Given the smooth terms, the GAM did not show indications of association between the spatial determinants and *Ehrlichia*-carrying ticks. Supplementary Figure S2 shows the habitat suitability map based on the GAM. Given a Cook's distance of 1.64, a potential influential outlier in the form of data from site 5038, was removed. Model assumptions were then met and there were no more indications for potential influential outliers (VIF Bio 18 and Bio 7: 1.37, Cook's distance of 0.84).

SUPPLEMENTARY TABLES

Supplementary Table S1. Latitude and longitude in World Geodetic System 1984 (WGS84) of each sampling site.

Site(ID)	WGS84Latitude	WGS84Longitude
5000	46.1442	11.9521
5001	46.1993037	12.1253363
5002	46.1825	12.195
5003	46.478346	12.448012
5004	46.158888	12.0573201
5005	46.032543	12.009134
5006	46.130056	12.310583
5009	46.106111	12.287778
5014	46.136444	12.367389
5017	46.0603207	11.913008
5018	46.0539569	12.2283882
5019	46.139861	12.351278
5026	46.29261	12.80602
5038	45.6415	11.12927
5041	45.98786	12.21527
5044	46.40963	13.18198
5074	46.092943	12.221204
5079	46.104861	12.441278
5080	46.067969	11.830914
5084	46.308424	12.010278
5091	46.1882798	12.0387767
5092	46.1774617	12.1896286

5093	46.2426104	12.3038316
5094	46.1697452	12.1151069
5095	46.1088134	12.2840926
5096	46.1367489	12.2593797

Supplementary Table S2. PCR systems used for tick-borne pathogen detection.

Specie	Target	Name	Sequence	Lenght (bp)	Use	Reference
Borrelia spp	23S rRNA	Bo_bu_sl_23S_F	GAGTCTTAAAAGGGCGATTTAGT	73	Screening by real-time PCR	[48]
		Bo_bu_sl-23S_R	CTTCAGCCTGGCCATAAATAG			
Borrelia spp	Fla	FlaB280F	GCAGTTCARTCAGGTAACGG	645	Nested-PCR and sequencing	[49]
		FlaRL	GCAATCATAGCCATTGCAGATTGT			
		flaB_737F	GCATCAACTGTRGTTGTAACATTAACAGG	407		
		FlaLL	ACATATTCAGATGCAGACAGAGGT			

Anaplasma spp	16S rRNA	Ana_spp_16S_F<br>Ana_spp_16S_R	CTTAGGGTTGTAAAACTCTTTCAG<br>CTTTAACTTACCAAACCGCCTAC	160	Screening by real-time PCR	[48]
A. phagocytophilum	msp2	An_ph_msp2_F<br>An_ph_msp2_R	GCTATGGAAGGCAGTGTTGG<br>GTCTTGAAGCGCTCGTAACC	77	Screening by real-time PCR	[50]
Anaplasma / Ehrlichia	16S rRNA	EHR1<br>EHR2<br>EHR3<br>EHR4	GAACGAACGCTGGCGGCAAGC<br>AGT AYC GRA CCA GAT AGC CGC<br>TGCATAGGAATCTACCTAGTAG<br>AGT AYC GRA CCA GAT AGC CGC	ND / 693<br>629 / 592	Nested-PCR and sequencing	[51]

Rickettsia spp.	gltA	Rick_spp_gltA_F	GTCGCAAATGTTCACGGTACTT	78	Screening by real-time PCR	[48]
		Rick_spp_gltA_R	TCTTCGTGCATTTCTTTCCATTG			
Rickettsia spp. 1	citrate synthase	Rsfg877	GGG GGC CTG CTC ACG GCG G	381	PCR and sequencing	[52]
		Rsfg1258	ATT GCA AAA AGT ACA GTG AAC A			
Rickettsia spp. 2	OmpB	Rc.rompB.4362p	GTCAGCGTTACTTCTTCGATGC	475	Nested-PCR and sequencing	[53]
		Rc.rompB.4,836n	CCGTACTCCATCTTAGCATCAG			
		Rc.rompB.4,496p	CCAATGGCAGGACTTAGCTACT	267		
		Rc.rompB.4,762n	AGGCTGGCTGATACACGGAGTAA			

Babesia/Theileria	18S rRNA	RLB-F2<br>RLB-R2	GACACAGGGAGGTAGTGACAA<br>CTAAGAATTTCACCTCTGACAGT	400	Screening by PCR and sequencing	[54]
Ixodes ricinus (Nucleic acid extraction control)	18S ribosomal RNA	F-16sIxodes<br>R-16sIxodes	AAAAAAATACTCTAGGGATAACAGCGTAA<br>ACCAAAAAAGAATCCTAATCCAACA	97	Screening by real-time PCR	[20]
TBEV	3' non-coding region	F-TBE 1<br>R-TBE 1	GGGCGGTTCTTGTTCTCC<br>ACACATCACCTCCTTGTCAGACT	67	Screening by real-time PCR	[20]
CCHFV	S segment	CCHFV NF<br>CCHFV NR	CAAGGGGTACCAAGAAAATGAAGAAGGC<br>GCCACAGGGATTGTTCCAAAGCAGAC	181	Screening by real-time PCR	[55]

Supplementary Table S3. Descriptive statistics tick data.

Statistics	Ticks Total	Larves	Nymphes	Males	Females
Mean	29.84	4.70	22.88	1.18	1.09
Standard Deviation	47.15	17.95	38.08	1.86	2.29
Median	9	0	7	0	0
First quartile	2	0	2	0	0
Third quartile	33	0	27	2	1
Minimum	1	0	0	0	0
Maximum	256	124	206	8	15
Range	255	124	206	8	15

n = 105 sampling moments

Supplementary Table S4. Presence of pathogens per tick life stage.

	Percentage of sampling moments (n/N)
No pathogen detected in any stadium	36% (35/98)
Pathogen presence solely in larvae stadium	3% (3/98)
Pathogen presence solely in nymph stadium	38% (37/98)
Pathogen presence solely in adult stadium	8% (8/98)
Pathogen presence in larvae and nymph stadium	2% (2/98)
Pathogen presence in larvae and adult stadium	0% (0/98)
Pathogen presence in nymph and adult stadium	8% (8/98)
Pathogen presence in all stadia (larvae, nymph, adult)	2% (2/98)

Supplementary Table S5. Presence of pathogens at each sampling site.

Site	WGS84		Percentage of sampling moments positive for			
(ID)	Latitude	Longitude	Any pathogen (n/N)	*Borrelia* spp. (n/N)	*Rickettsia* spp. (n/N)	*Anaplasma/Ehrlichia* spp. (n/N)
5000	46.1442	11.9521	38% (3/8)	13% (1/8)	13% (1/8)	38% (3/8)
5001	46.1993037	12.1253363	90% (9/10)	60% (6/10)	50% (5/10)	50% (5/10)
5002	46.1825	12.195	38% (5/13)	31 % (4/13)	8% (1/13)	8% (1/13)
5003	46.478346	12.448012	66% (2/3)	66% (2/3)	0% (0/3)	0% (0/3)
5004	46.158888	12.0573201	83% (10/12)	33% (4/12)	50% (6/12)	50% (6/12)
5005	46.032543	12.009134	100% (1/1)	100% (1/1)	100% (1/1)	0% (0/1)
5006	46.130056	12.310583	33% (1/3)	33% (1/3)	0% (0/3)	0% (0/3)
5009	46.106111	12.287778	33% (2/6)	17% (1/6)	0% (0/6)	33% (1/6)
5014	46.136444	12.367389	50% (1/2)	50% (1/2)	0% (0/2)	0% (0/2)
5017	46.0603207	11.913008	50% (1/2)	50% (1/2)	0% (0/2)	0% (0/2)
5018	46.0539569	12.2283882	55% (6/11)	27% (3/11)	45% (5/11)	18% (2/11)
5019	46.139861	12.351278	100% (3/3)	67% (2/3)	33% (1/3)	0% (0/3)

5026	46.29261	12.80602	100% (1/1)	100% (1/1)	0% (0/1)	100% (1/1)
5038	45.6415	11.12927	100% (1/1)	100% (1/1)	0% (0/1)	100% (1/1)
5041	45.98786	12.21527	100% (1/1)	100% (1/1)	100% (1/1)	0% (0/1)
5044	46.40963	13.18198	100% (1/1)	100% (1/1)	100% (1/1)	100% (1/1)
5074	46.092943	12.221204	100% (4/4)	50% (2/4)	75% (3/4)	25% (1/4)
5079	46.104861	12.441278	0% (0/1)	0% (0/1)	0% (0/1)	0% (0/1)
5080	46.067969	11.830914	0% (0/1)	0% (0/1)	0% (0/1)	0% (0/1)
5084	46.308424	12.010278	67% (2/3)	33% (1/3)	33% (1/3)	0% (0/3)
5091	46.1882798	12.0387767	100% (1/1)	0% (0/1)	100% (1/1)	0% (0/1)
5092	46.1774617	12.1896286	100% (4/4)	25% (1/4)	75% (3/4)	50% (2/4)
*5093	46.2426104	12.3038316				
5094	46.1697452	12.1151069	66% (2/3)	33% (1/3)	66% (2/3)	0% (0/3)
5095	46.1088134	12.2840926	50% (1/2)	50% (1/2)	50% (1/2)	50% (1/2)
5096	46.1367489	12.2593797	100% (1/1)	0% (0/1)	100% (1/1)	0% (0/1)

* Pathogen detection did not take place after data collection at this specific site

Supplementary Table S6. Descriptive statistics spatial determinants in North-East Italy.

Spatial Determinants North-East Italy						
Statistics	Bio 1	Bio 2	Bio 3	Bio 4	Bio 5	Bio 6
Mean	9.78	9.85	30.60	811.50	26.39	-5.54
Standard Deviation	4.90	2.15	4.23	42.46	5.88	3.47
Median	10.78	9.58	31.15	804.10	26.56	-4.38
First quartile	5.98	7.95	27.13	773.58	21.34	-7.93
Third quartile	14.50	12.14	34.63	853.68	32.31	-2.58
Minimum	-5.49	3.70	14.49	712.55	8.48	-17.09
Maximum	15.30	13.37	37.3	900.72	34.38	-0.18
Range	20.78	9.67	22.81	188.18	25.90	16.91

Spatial Determinants North-East Italy						
Statistics	Bio 7	Bio 8	Bio 9	Bio 10	Bio 11	Bio 12
Mean	31.93	13.56	3.18	19.64	-0.28	41.65
Standard Deviation	2.80	4.22	5.91	5.10	4.37	13.68
Median	31.10	13.40	3.41	20.33	0.98	39.35
First quartile	29.39	10.50	-1.33	15.49	-3.39	32.58
Third quartile	34.89	16.90	7.94	24.65	3.68	47.68
Minimum	25.42	0.57	-11.44	3.74	-14.58	21.34
Maximum	37.09	23.40	17.85	25.98	4.71	101.34
Range	11.67	22.90	29.29	22.24	19.29	80.00

Spatial Determinants North-East Italy						
Statistics	Bio 13	Bio 14	Bio 15	Bio 16	Bio 17	Bio 18
Mean	8.63	0.27	55.18	16.85	4.15	12.18
Standard Deviation	3.43	0.12	5.80	5.54	1.73	4.10
Median	7.86	0.25	54.82	16.53	3.75	12.48
First quartile	6.37	0.19	50.74	12.87	2.93	8.67
Third quartile	9.83	0.32	58.59	19.60	4.84	15.27
Minimum	4.14	0.05	43.01	8.38	1.59	5.04
Maximum	21.08	0.88	74.56	38.47	12.84	22.96
Range	16.94	0.83	31.55	30.09	11.26	17.92

Spatial Determinants North-East Italy						
Statistics	Bio 19	DTM	N*_Autumn	N*_Winter	N*_Spring	N*_Summer
Mean	8.07	846.47	0.47	0.28	0.37	0.57
Standard Deviation	4.27	838.93	0.20	0.19	0.22	0.20
Median	6.72	645.75	0.52	0.30	0.42	0.63
First quartile	4.84	33.50	0.36	0.16	0.23	0.51
Third quartile	9.89	1509.24	0.63	0.42	0.55	0.71
Minimum	2.65	-6.00	-0.50	-0.59	-0.40	-0.43
Maximum	29.65	3831.77	0.82	0.81	0.83	0.87
Range	27.00	3837.77	1.32	1.40	1.23	1.30

*N = NDVI

Frequency Table CLC North-East Italy			
Grid_Code	Frequency	Percentage	Cumulative percentage
11	2244	3.64	3.64
12	706	1.15	4.79
13	72	0.12	4.91
14	78	0.13	5.03
21	15213	24.71	29.74
22	1729	2.81	32.55

Supplementary Table S7. Descriptive statistics spatial determinants at the sampling sites.

Statistics	Bio 1	Bio 2	Bio 3	Bio 4	Bio 5	Bio 6
	Spatial Determinants Sampling Sites					
Mean	10.45	9.61	31.20	789.30	26.18	-4.67
Standard Deviation	1.18	0.75	1.25	26.55	1.62	0.77
Median	10.60	9.61	31.22	787.90	26.05	-4.75
First quartile	9.58	8.96	30.05	769.30	25.04	-5.22
Third quartile	11.50	10.17	32.20	807.70	27.31	-4.01
Minimum	8.17	8.56	29.18	745.00	23.41	-6.10
Maximum	12.37	10.98	33.25	850.40	28.57	-3.18
Range	4.20	2.42	4.07	105.40	5.16	2.92

Statistics	Bio 7	Bio 8	Bio 9	Bio 10	Bio 11	Bio 12
	Spatial Determinants Sampling Sites					
Mean	31.00	13.23	3.05	20.01	0.61	55.42
Standard Deviation	1.70	2.70	2.36	1.32	0.95	10.13
Median	30.77	13.46	2.56	20.04	0.77	52.89
First quartile	29.74	12.04	1.52	19.03	-0.11	50.62
Third quartile	31.29	15.26	3.23	21.17	1.38	55.42
Minimum	28.99	5.66	0.49	17.64	-1.18	44.30
Maximum	36.66	17.18	11.73	21.80	2.76	90.8
Range	7.67	11.51	11.24	4.16	3.94	46.50

Spatial Determinants Sampling Sites						
Statistics	Bio 13	Bio 14	Bio 15	Bio 16	Bio 17	Bio 18
Mean	12.10	0.24	60.8	21.45	5.57	14.51
Standard Deviation	2.68	0.09	3.44	4.28	1.42	1.53
Median	11.59	0.21	59.87	20.4	5.31	14.46
First quartile	10.40	0.20	58.41	19.65	4.96	13.50
Third quartile	12.37	0.24	62.49	21.29	5.52	15.16
Minimum	9.48	0.17	56.61	17.63	4.00	11.28
Maximum	20.14	0.64	70.63	35.95	10.90	19.34
Range	10.65	0.46	14.02	18.32	6.9	8.06

Spatial Determinants Sampling Sites						
Statistics	Bio 19	DTM	N*_Autumn	N*_Winter	N*_Spring	N*_Summer
Mean	10.89	707.12	0.62	0.24	0.49	0.72
Standard Deviation	3.64	563.95	0.12	0.18	0.13	0.14
Median	9.55	551.33	0.65	0.27	0.53	0.76
First quartile	8.92	215.94	0.6	0.11	0.43	0.71
Third quartile	10.99	1164.07	0.68	0.37	0.57	0.78
Minimum	6.78	38.24	0.21	-0.09	0.15	0.26
Maximum	23.63	1957.67	0.77	0.49	0.74	0.86
Range	16.84	1919.43	0.55	0.58	0.59	0.59

*N = NDVI

Frequency Table CLC Sampling Sites			
Grid_Code	Frequency	Percentage	Cumulative percentage
11	0	0.00	0.00
12	0	0.00	0.00
13	0	0.00	0.00
14	0	0.00	0.00
21	2	7.69	7.69
22	1	3.85	11.54
23	0	0.00	11.54
24	0	0.00	11.54
31	14	53.85	65.38
32	2	7.69	73.08
33	6	23.08	96.15
41	0	0.00	96.15
42	0	0.00	96.15
51	0	0.00	96.15
52	1	3.85	100
Total	26	100	100

Supplementary Table S8. Results univariable regression analysis Pathogens.

GAM Pathogens		
Spatial Determinant	p-value	Model fit (R-squared)
Bio 1	0	0.11
Bio 2	0.22	0.08
Bio 3	0.35	0
Bio 4	0.03	0.13
Bio 5	0.02	0.09
Bio 6	0	0.11
Bio 7	0.8	0.01
Bio 8	0.09	0.03
Bio 9	0.13	0.06
Bio 10	0	0.11
Bio 11	0	0.08
Bio 12	0.6	0.01
Bio 13	0.36	0.04
Bio 14	0.35	0
Bio 15	0.34	0.05
Bio 16	0.12	0.11
Bio 17	0.33	0.05
Bio 18	0.75	0.01
Bio 19	0.13	0.07
NDVI_Autumn	0.21	0.05

NDVI_Winter	0.01	0.08
NDVI_Spring	0.05	0.16
NDVI_Summer	0.29	0.05
DTM	0.24	0
CLC 2.1	0.48	0.01
CLC 2.2	0.99	0.01
CLC 3.1	0.2	0.01
CLC 3.2	0.99	0.01
CLC 3.3	0.06	0.01
CLC 5.2	0.99	0.01

Supplementary Table S9. Results univariable regression analysis Borrelia spp.

GAM *Borrelia* spp.		
Spatial Determinant	p-value	Model fit (R-squared)
Bio 1	0.02	0.05
Bio 2	0.09	0.02
Bio 3	0.13	0.01
Bio 4	0.1	0.02
Bio 5	0.02	0.05
Bio 6	0.06	0.02
Bio 7	0.05	0.03
Bio 8	0.42	0
Bio 9	0.41	0.05
Bio 10	0.02	0.05
Bio 11	0.05	0.03
Bio 12	0.46	0.06
Bio 13	0.55	0.03
Bio 14	0.15	0.03
Bio 15	0.61	0.01
Bio 16	0.6	0.03
Bio 17	0.16	0.02
Bio 18	0.86	0.02
Bio 19	0.28	0.03
NDVI_Autumn	0.3	0.02

NDVI_Winter	0.95	-0.01
NDVI_Spring	0.3	0.03
NDVI_Summer	0.22	0.01
DTM	0.58	-0.01
CLC 2.1	0.18	0.02
CLC 2.2	0.99	0.02
CLC 3.1	0.57	0.02
CLC 3.2	0.99	0.02
CLC 3.3	0.35	0.02
CLC 5.2	0.99	0.02

Supplementary Table S10. Results univariable regression analysis Rickettsia spp.

GAM *Rickettsia spp.*		
Spatial Determinant	p-value	Model fit (R-squared)
Bio 1	0.01	0.07
Bio 2	0.98	-0.01
Bio 3	0.61	0.02
Bio 4	0.03	0.14
Bio 5	0.1	0.04
Bio 6	0	0.1
Bio 7	0.76	0
Bio 8	0.02	0.05
Bio 9	0.03	0.05
Bio 10	0.03	0.06
Bio 11	0.09	0.07
Bio 12	0.41	0.02
Bio 13	0.76	-0.01
Bio 14	0.5	0.02
Bio 15	0.31	0.05
Bio 16	0.17	0.11
Bio 17	0.15	0.07
Bio 18	0.28	0
Bio 19	0.06	0.12
NDVI_Autumn	0.19	0.09
NDVI_Winter	0.02	0.08

NDVI_Spring	0.14	0.09
NDVI_Summer	0.15	0.07
DTM	0.06	0.31
CLC 2.1	0.07	0.01
CLC 2.2	0.99	0.01
CLC 3.1	0.27	0.01
CLC 3.2	0.27	0.01
CLC 3.3	0.1	0.01
CLC 5.2	0.99	0.01

Supplementary Table S11. Results univariable regression analysis Ehrlichia spp.

GAM *Ehrlichia spp.*		
Spatial Determinant	p-value	Model fit (R-squared)
Bio 1	0.6	0.01
Bio 2	0.32	0.1
Bio 3	0.24	0.07
Bio 4	0.21	0.15
Bio 5	0.31	0.01
Bio 6	0.08	0.08
Bio 7	0.08	0.07
Bio 8	0.42	0.12
Bio 9	0.24	0.01
Bio 10	0.41	0.03
Bio 11	0.71	-0.01
Bio 12	0.04	0.07
Bio 13	0.21	0.07
Bio 14	0.47	0.07
Bio 15	0.36	0.15
Bio 16	0.03	0.07
Bio 17	0.04	0.07
Bio 18	0.11	0.09
Bio 19	0.41	0.18
NDVI_Autumn	0.37	0.02
NDVI_Winter	0.1	0.08

NDVI_Spring	0.65	0
NDVI_Summer	0.44	0.02
DTM	0.59	0.12
CLC 2.1	0.18	-0.01
CLC 2.2	0.99	-0.01
CLC 3.1	1	-0.01
CLC 3.2	0.5	-0.01
CLC 3.3	0.88	-0.01
CLC 5.2	0.99	-0.01

SUPPLEMENTARY FIGURES

Supplementary Figure S1. Correlation matrix spatial determinants.

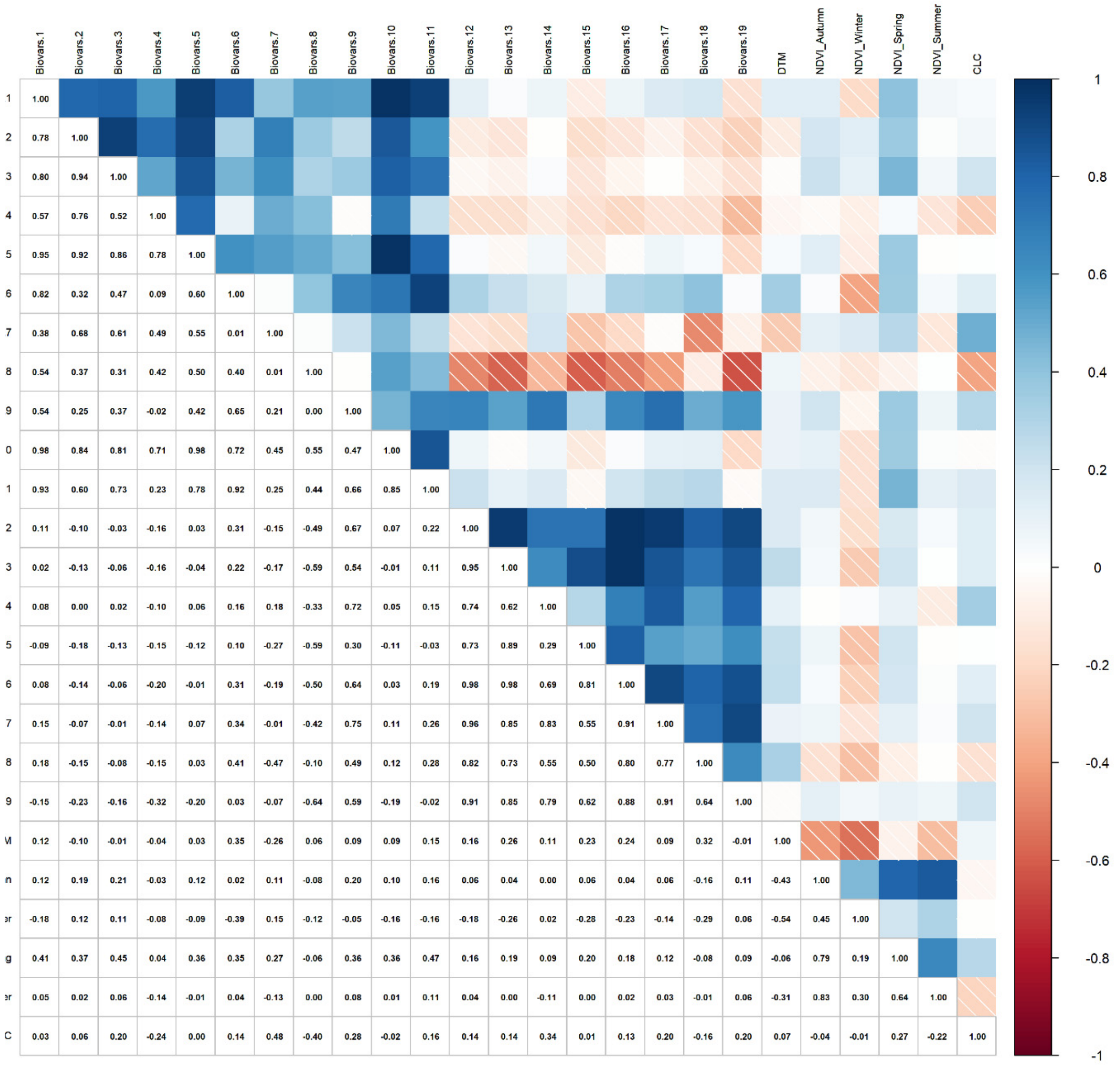

Supplementary Figure S2. Habitat suitability maps for North-East Italy from GAM. Greener colours indicate a better habitat suitability, for (a) probability of presence of all pathogen-carrying ticks, (b) probability of presence of Borrelia-carrying ticks, (c) probability of presence of Rickettsia-carrying ticks, and (d) probability of presence of Ehrlichia-carrying ticks.

(a)

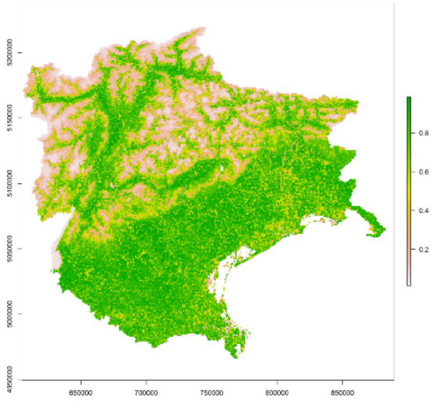

(b)

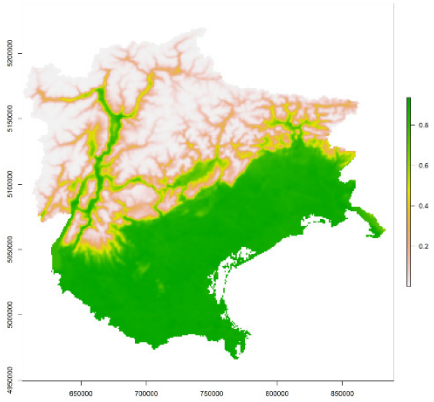

(c)

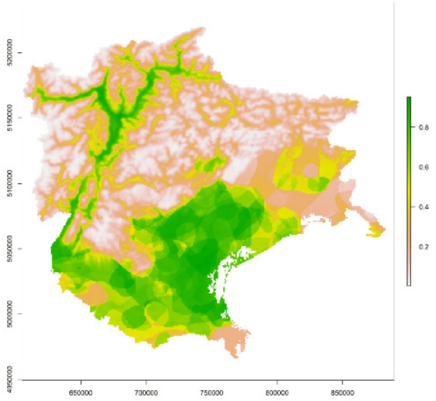
5200000
5150000
5100000
5050000
5000000
4950000
650000
700000
750000
800000
850000
0.8
0.6
0.4
0.2

(d)

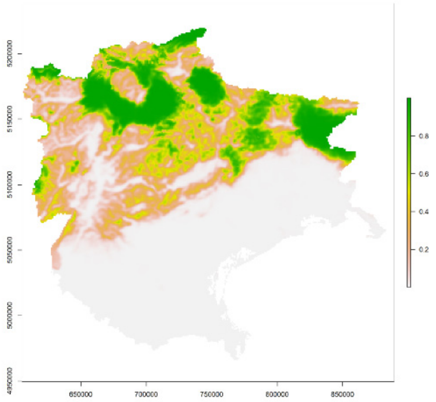
5200000
5150000
5100000
5050000
5000000
4950000
650000
700000
750000
800000
850000
0.8
0.6
0.4
0.2

SUPPLEMENTARY DETAILS ON MAXENT MODELLING

Results Maxent model for probability of presence of pathogen-carrying ticks

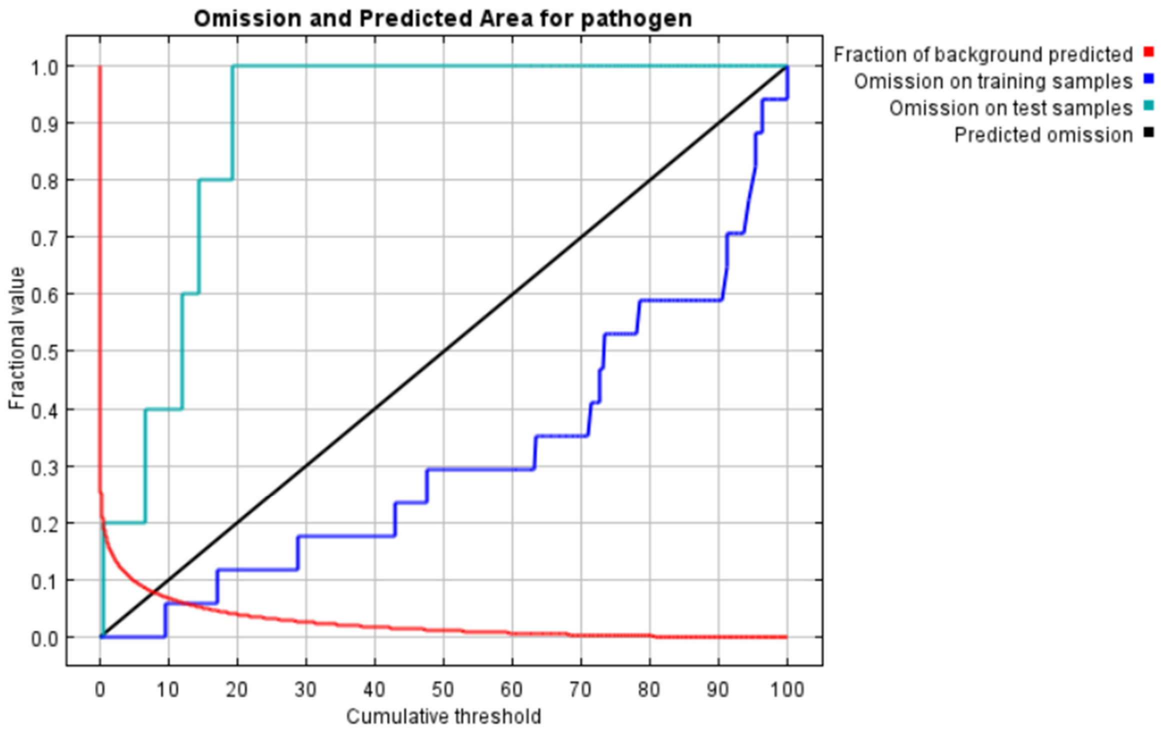

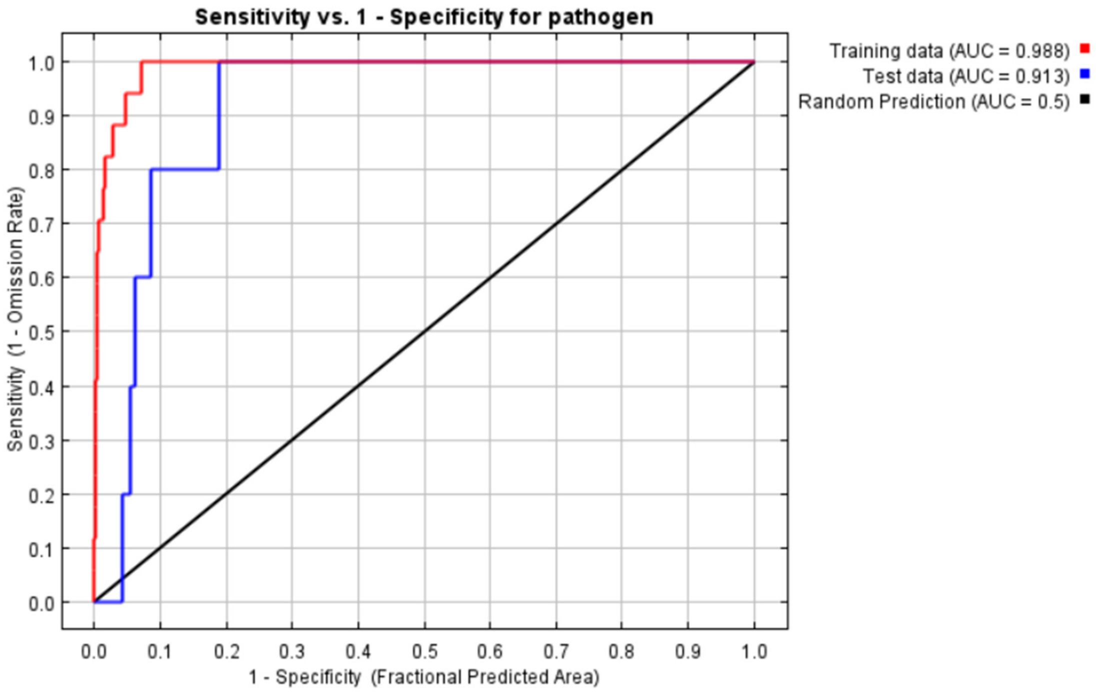

Variable	Percent contribution	Permutation importance
DTM_TRIV_1km	30.3	50.2
MEAN_BIOVARS_TRIV_2015-20_1km_13	29.2	1.8
MEAN_NDVI_SUMMER_2015-20_1km	12.6	4.5
CLC_TRIV_1km_lvl_2	10.5	2.2
MEAN_BIOVARS_TRIV_2015-20_1km_11	5.7	39.6
MEAN_BIOVARS_TRIV_2015-20_1km_17	2.9	0
MEAN_BIOVARS_TRIV_2015-20_1km_14	2.9	0.2
MEAN_BIOVARS_TRIV_2015-20_1km_04	2.1	0.1
MEAN_BIOVARS_TRIV_2015-20_1km_08	2.1	0.6
MEAN_BIOVARS_TRIV_2015-20_1km_01	1	0
MEAN_BIOVARS_TRIV_2015-20_1km_19	0.5	0.4
MEAN_BIOVARS_TRIV_2015-20_1km_15	0.1	0.2
MEAN_BIOVARS_TRIV_2015-20_1km_12	0	0
MEAN_NDVI_SPRING_2015-20_1km	0	0.1
MEAN_BIOVARS_TRIV_2015-20_1km_03	0	0
MEAN_BIOVARS_TRIV_2015-20_1km_02	0	0
MEAN_BIOVARS_TRIV_2015-20_1km_18	0	0
MEAN_BIOVARS_TRIV_2015-20_1km_16	0	0
MEAN_NDVI_AUTUMN_2015-20_1km	0	0
MEAN_NDVI_WINTER_2015-20_1km	0	0
MEAN_BIOVARS_TRIV_2015-20_1km_10	0	0
MEAN_BIOVARS_TRIV_2015-20_1km_09	0	0
MEAN_BIOVARS_TRIV_2015-20_1km_07	0	0
MEAN_BIOVARS_TRIV_2015-20_1km_06	0	0
MEAN_BIOVARS_TRIV_2015-20_1km_05	0	0

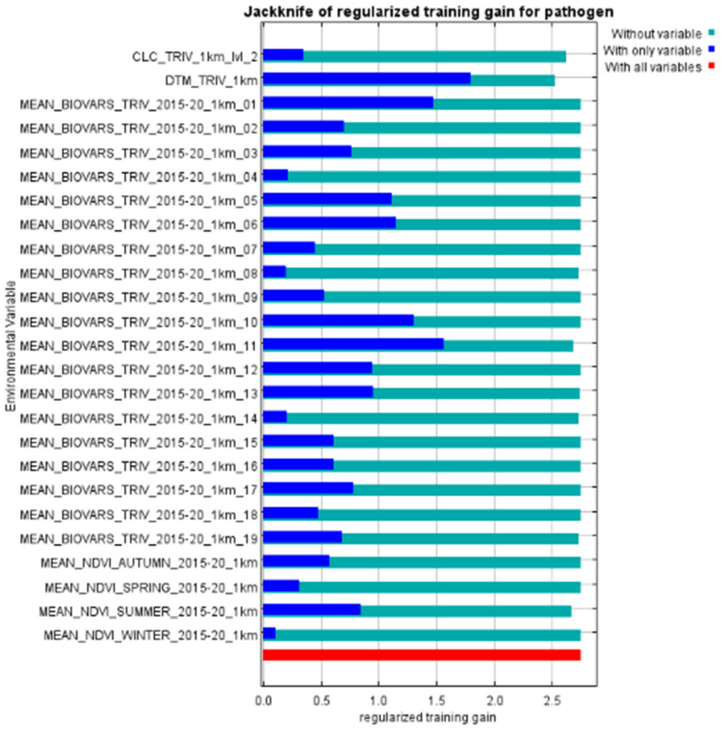

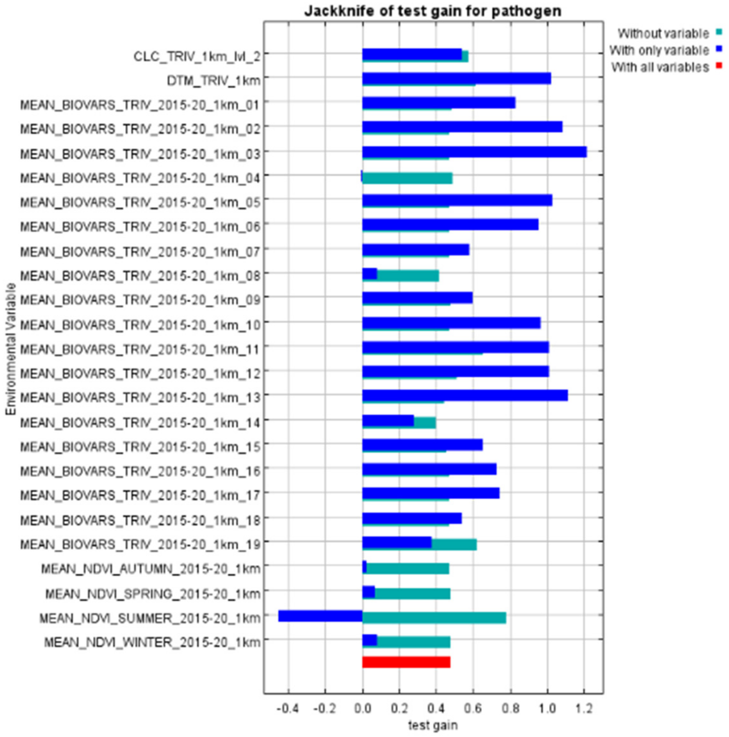

Jackknife of test gain for pathogen
Without variable
With only variable
With all variables
CLC_TRIV_1km_M_2
DTM_TRIV_1km
MEAN_BIOVARS_TRIV_2015-20_1km_01
MEAN_BIOVARS_TRIV_2015-20_1km_02
MEAN_BIOVARS_TRIV_2015-20_1km_03
MEAN_BIOVARS_TRIV_2015-20_1km_04
MEAN_BIOVARS_TRIV_2015-20_1km_05
MEAN_BIOVARS_TRIV_2015-20_1km_06
MEAN_BIOVARS_TRIV_2015-20_1km_07
MEAN_BIOVARS_TRIV_2015-20_1km_08
MEAN_BIOVARS_TRIV_2015-20_1km_09
MEAN_BIOVARS_TRIV_2015-20_1km_10
MEAN_BIOVARS_TRIV_2015-20_1km_11
MEAN_BIOVARS_TRIV_2015-20_1km_12
MEAN_BIOVARS_TRIV_2015-20_1km_13
MEAN_BIOVARS_TRIV_2015-20_1km_14
MEAN_BIOVARS_TRIV_2015-20_1km_15
MEAN_BIOVARS_TRIV_2015-20_1km_16
MEAN_BIOVARS_TRIV_2015-20_1km_17
MEAN_BIOVARS_TRIV_2015-20_1km_18
MEAN_BIOVARS_TRIV_2015-20_1km_19
MEAN_NDVI_AUTUMN_2015-20_1km
MEAN_NDVI_SPRING_2015-20_1km
MEAN_NDVI_SUMMER_2015-20_1km
MEAN_NDVI_WINTER_2015-20_1km
Environmental Variable
-0.4 -0.2 0.0 0.2 0.4 0.6 0.8 1.0 1.2
test gain

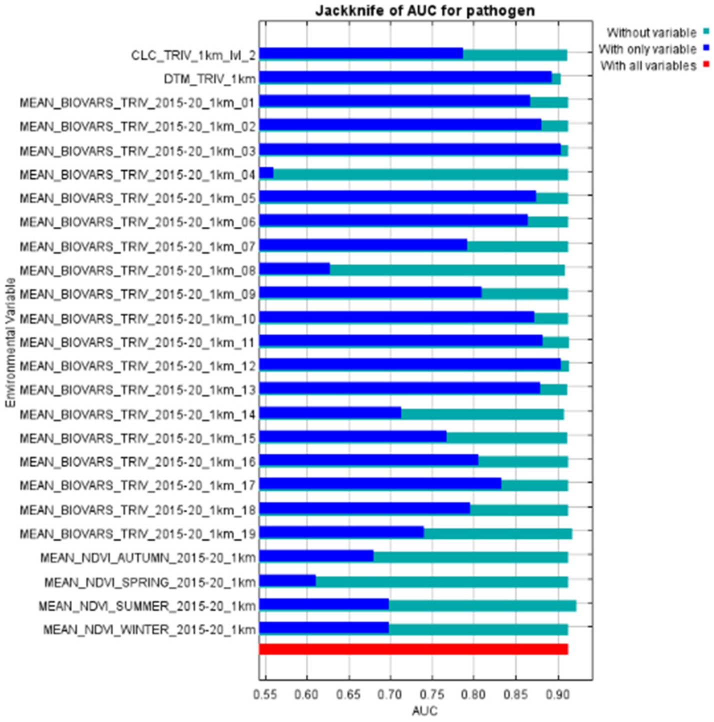

Jackknife of AUC for pathogen
Without variable
With only variable
With all variables
CLC_TRIV_1km_M_2
DTM_TRIV_1km
MEAN_BIOVARS_TRIV_2015-20_1km_01
MEAN_BIOVARS_TRIV_2015-20_1km_02
MEAN_BIOVARS_TRIV_2015-20_1km_03
MEAN_BIOVARS_TRIV_2015-20_1km_04
MEAN_BIOVARS_TRIV_2015-20_1km_05
MEAN_BIOVARS_TRIV_2015-20_1km_06
MEAN_BIOVARS_TRIV_2015-20_1km_07
MEAN_BIOVARS_TRIV_2015-20_1km_08
MEAN_BIOVARS_TRIV_2015-20_1km_09
MEAN_BIOVARS_TRIV_2015-20_1km_10
MEAN_BIOVARS_TRIV_2015-20_1km_11
MEAN_BIOVARS_TRIV_2015-20_1km_12
MEAN_BIOVARS_TRIV_2015-20_1km_13
MEAN_BIOVARS_TRIV_2015-20_1km_14
MEAN_BIOVARS_TRIV_2015-20_1km_15
MEAN_BIOVARS_TRIV_2015-20_1km_16
MEAN_BIOVARS_TRIV_2015-20_1km_17
MEAN_BIOVARS_TRIV_2015-20_1km_18
MEAN_BIOVARS_TRIV_2015-20_1km_19
MEAN_NDVI_AUTUMN_2015-20_1km
MEAN_NDVI_SPRING_2015-20_1km
MEAN_NDVI_SUMMER_2015-20_1km
MEAN_NDVI_WINTER_2015-20_1km
Environmental Variable
0.55 0.60 0.65 0.70 0.75 0.80 0.85 0.90
AUC

Results Maxent model for probability of presence of *Borrelia*-carrying ticks

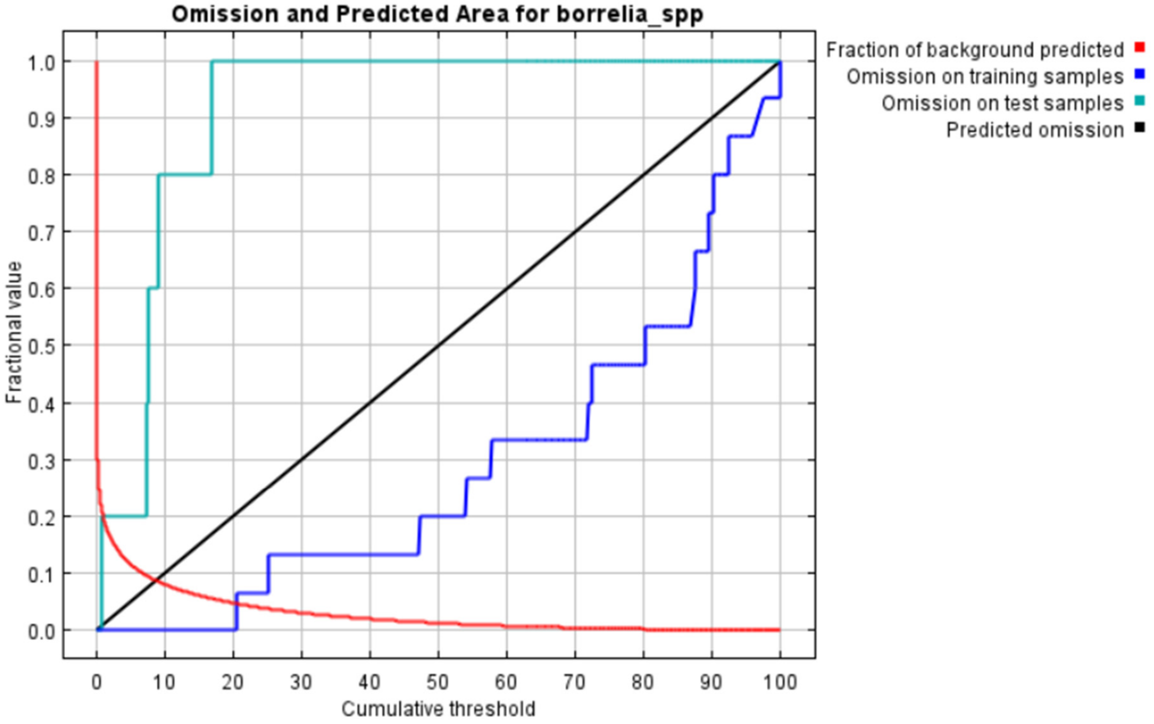

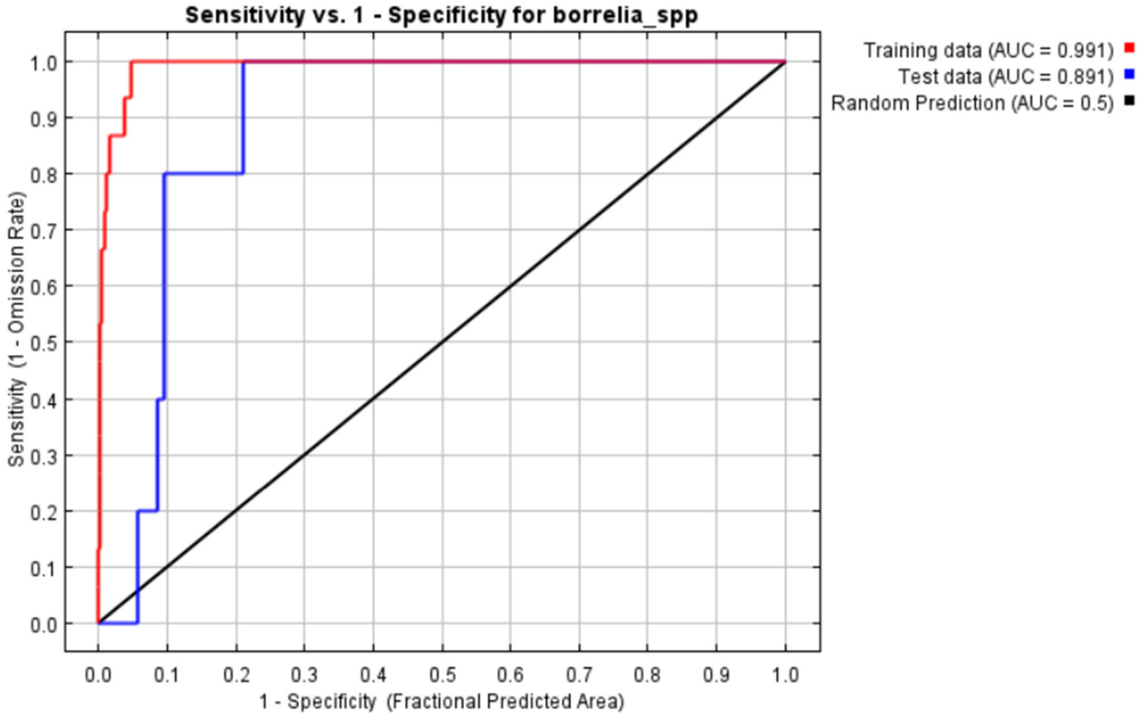

Variable	Percent contribution	Permutation importance
MEAN_BIOVARS_TRIV_2015-20_1km_13	34.2	6.2
DTM_TRIV_1km	30.3	52.7
MEAN_NDVI_SUMMER_2015-20_1km	13.5	7.3
CLC_TRIV_1km_lvl_2	8.3	0.6
MEAN_BIOVARS_TRIV_2015-20_1km_08	6.6	3.3
MEAN_BIOVARS_TRIV_2015-20_1km_14	3.1	0.4
MEAN_BIOVARS_TRIV_2015-20_1km_11	0.7	25.1
MEAN_BIOVARS_TRIV_2015-20_1km_04	0.7	0
MEAN_BIOVARS_TRIV_2015-20_1km_17	0.7	0
MEAN_BIOVARS_TRIV_2015-20_1km_15	0.7	0.3
MEAN_NDVI_AUTUMN_2015-20_1km	0.5	0
MEAN_BIOVARS_TRIV_2015-20_1km_19	0.4	4.1
MEAN_BIOVARS_TRIV_2015-20_1km_01	0.2	0
MEAN_NDVI_SPRING_2015-20_1km	0	0
MEAN_BIOVARS_TRIV_2015-20_1km_09	0	0
MEAN_BIOVARS_TRIV_2015-20_1km_16	0	0
MEAN_BIOVARS_TRIV_2015-20_1km_07	0	0
MEAN_BIOVARS_TRIV_2015-20_1km_06	0	0
MEAN_BIOVARS_TRIV_2015-20_1km_05	0	0
MEAN_BIOVARS_TRIV_2015-20_1km_18	0	0
MEAN_BIOVARS_TRIV_2015-20_1km_03	0	0
MEAN_BIOVARS_TRIV_2015-20_1km_02	0	0
MEAN_BIOVARS_TRIV_2015-20_1km_12	0	0
MEAN_NDVI_WINTER_2015-20_1km	0	0
MEAN_BIOVARS_TRIV_2015-20_1km_10	0	0

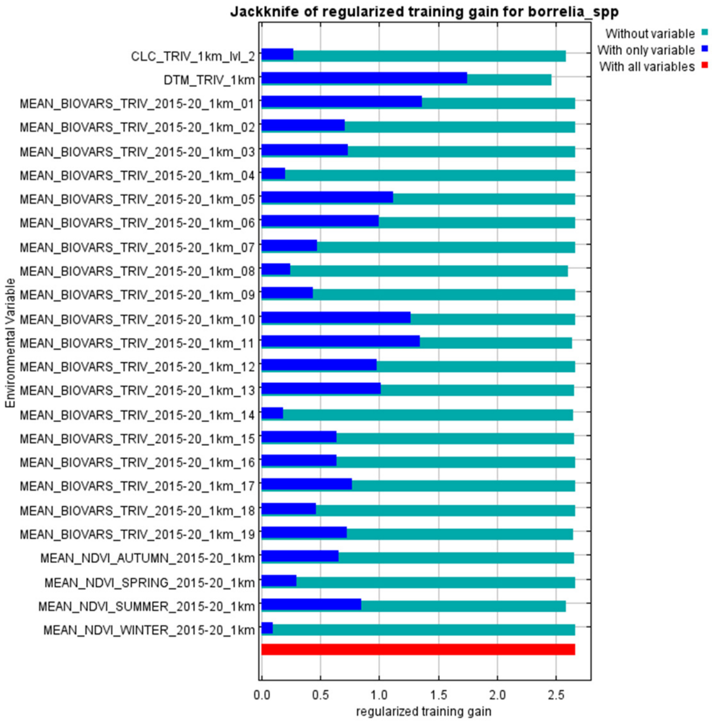

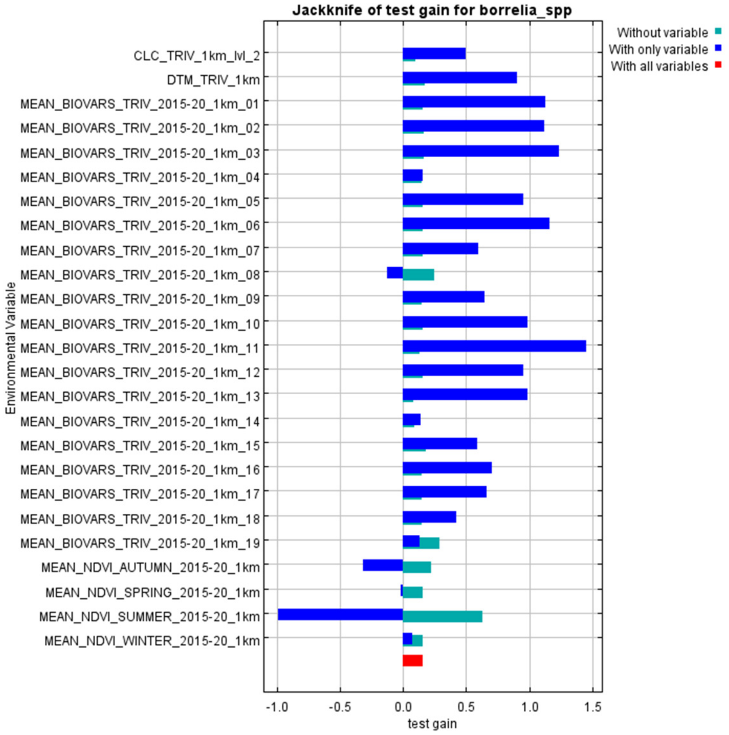

Jackknife of test gain for borrelia_spp
Without variable
With only variable
With all variables
CLC_TRIV_1km_lvl_2
DTM_TRIV_1km
MEAN_BIOVARS_TRIV_2015-20_1km_01
MEAN_BIOVARS_TRIV_2015-20_1km_02
MEAN_BIOVARS_TRIV_2015-20_1km_03
MEAN_BIOVARS_TRIV_2015-20_1km_04
MEAN_BIOVARS_TRIV_2015-20_1km_05
MEAN_BIOVARS_TRIV_2015-20_1km_06
MEAN_BIOVARS_TRIV_2015-20_1km_07
MEAN_BIOVARS_TRIV_2015-20_1km_08
MEAN_BIOVARS_TRIV_2015-20_1km_09
MEAN_BIOVARS_TRIV_2015-20_1km_10
MEAN_BIOVARS_TRIV_2015-20_1km_11
MEAN_BIOVARS_TRIV_2015-20_1km_12
MEAN_BIOVARS_TRIV_2015-20_1km_13
MEAN_BIOVARS_TRIV_2015-20_1km_14
MEAN_BIOVARS_TRIV_2015-20_1km_15
MEAN_BIOVARS_TRIV_2015-20_1km_16
MEAN_BIOVARS_TRIV_2015-20_1km_17
MEAN_BIOVARS_TRIV_2015-20_1km_18
MEAN_BIOVARS_TRIV_2015-20_1km_19
MEAN_NDVI_AUTUMN_2015-20_1km
MEAN_NDVI_SPRING_2015-20_1km
MEAN_NDVI_SUMMER_2015-20_1km
MEAN_NDVI_WINTER_2015-20_1km
Environmental Variable
-1.0
-0.5
0.0
0.5
1.0
1.5
test gain

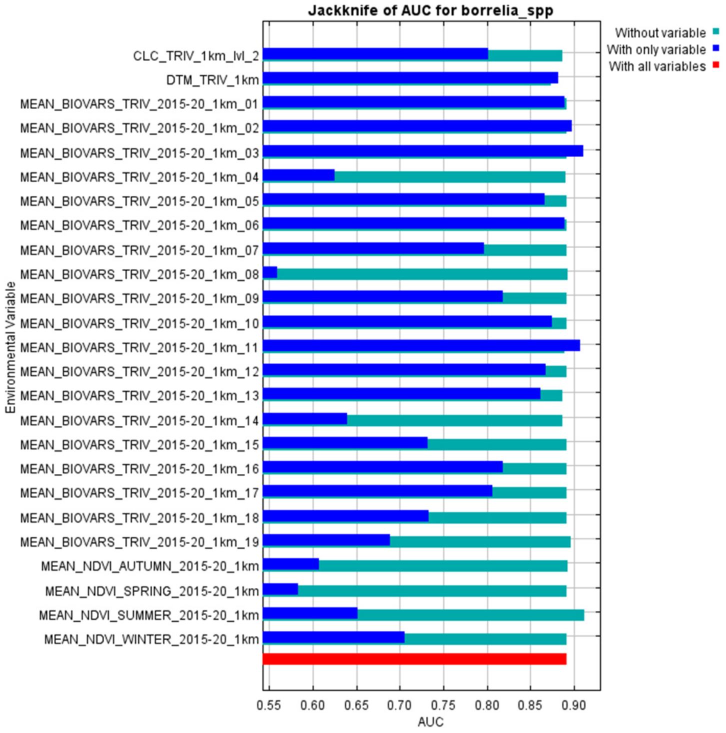

Jackknife of AUC for borrelia_spp
Without variable
With only variable
With all variables
CLC_TRIV_1km_lvl_2
DTM_TRIV_1km
MEAN_BIOVARS_TRIV_2015-20_1km_01
MEAN_BIOVARS_TRIV_2015-20_1km_02
MEAN_BIOVARS_TRIV_2015-20_1km_03
MEAN_BIOVARS_TRIV_2015-20_1km_04
MEAN_BIOVARS_TRIV_2015-20_1km_05
MEAN_BIOVARS_TRIV_2015-20_1km_06
MEAN_BIOVARS_TRIV_2015-20_1km_07
MEAN_BIOVARS_TRIV_2015-20_1km_08
MEAN_BIOVARS_TRIV_2015-20_1km_09
MEAN_BIOVARS_TRIV_2015-20_1km_10
MEAN_BIOVARS_TRIV_2015-20_1km_11
MEAN_BIOVARS_TRIV_2015-20_1km_12
MEAN_BIOVARS_TRIV_2015-20_1km_13
MEAN_BIOVARS_TRIV_2015-20_1km_14
MEAN_BIOVARS_TRIV_2015-20_1km_15
MEAN_BIOVARS_TRIV_2015-20_1km_16
MEAN_BIOVARS_TRIV_2015-20_1km_17
MEAN_BIOVARS_TRIV_2015-20_1km_18
MEAN_BIOVARS_TRIV_2015-20_1km_19
MEAN_NDVI_AUTUMN_2015-20_1km
MEAN_NDVI_SPRING_2015-20_1km
MEAN_NDVI_SUMMER_2015-20_1km
MEAN_NDVI_WINTER_2015-20_1km
Environmental Variable
0.55
0.60
0.65
0.70
0.75
0.80
0.85
0.90
AUC

Results Maxent model for probability of presence of *Rickettsia*-carrying ticks

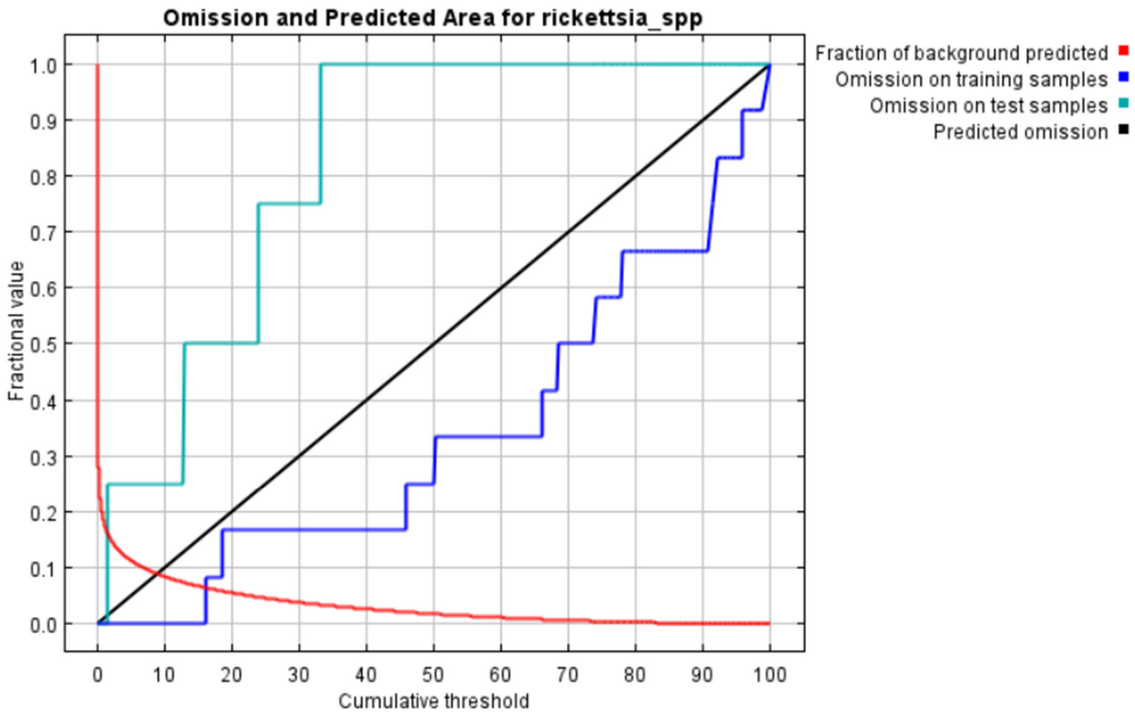

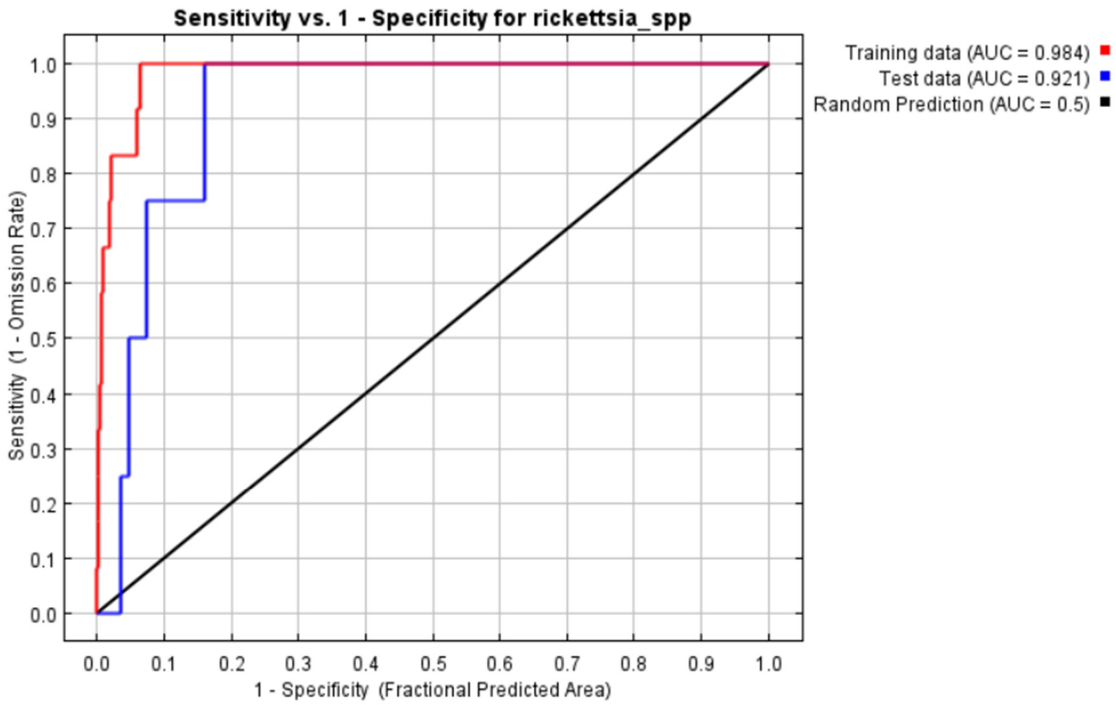

Variable	Percent contribution	Permutation importance
MEAN_NDVI_SUMMER_2015-20_1km	49.8	27.6
MEAN_BIOVARS_TRIV_2015-20_1km_11	27.6	38.3
DTM_TRIV_1km	10.5	25.9
CLC_TRIV_1km_lvl_2	5.1	1.1
MEAN_BIOVARS_TRIV_2015-20_1km_12	4	0
MEAN_NDVI_SPRING_2015-20_1km	1.3	3.8
MEAN_BIOVARS_TRIV_2015-20_1km_08	0.7	0
MEAN_BIOVARS_TRIV_2015-20_1km_14	0.5	0
MEAN_BIOVARS_TRIV_2015-20_1km_18	0.3	0.3
MEAN_BIOVARS_TRIV_2015-20_1km_07	0.1	0
MEAN_BIOVARS_TRIV_2015-20_1km_05	0.1	3
MEAN_BIOVARS_TRIV_2015-20_1km_06	0	0
MEAN_BIOVARS_TRIV_2015-20_1km_19	0	0
MEAN_BIOVARS_TRIV_2015-20_1km_17	0	0
MEAN_BIOVARS_TRIV_2015-20_1km_16	0	0
MEAN_BIOVARS_TRIV_2015-20_1km_15	0	0
MEAN_BIOVARS_TRIV_2015-20_1km_13	0	0
MEAN_BIOVARS_TRIV_2015-20_1km_10	0	0
MEAN_BIOVARS_TRIV_2015-20_1km_09	0	0
MEAN_BIOVARS_TRIV_2015-20_1km_04	0	0
MEAN_BIOVARS_TRIV_2015-20_1km_03	0	0
MEAN_BIOVARS_TRIV_2015-20_1km_02	0	0
MEAN_BIOVARS_TRIV_2015-20_1km_01	0	0
MEAN_NDVI_WINTER_2015-20_1km	0	0
MEAN_NDVI_AUTUMN_2015-20_1km	0	0

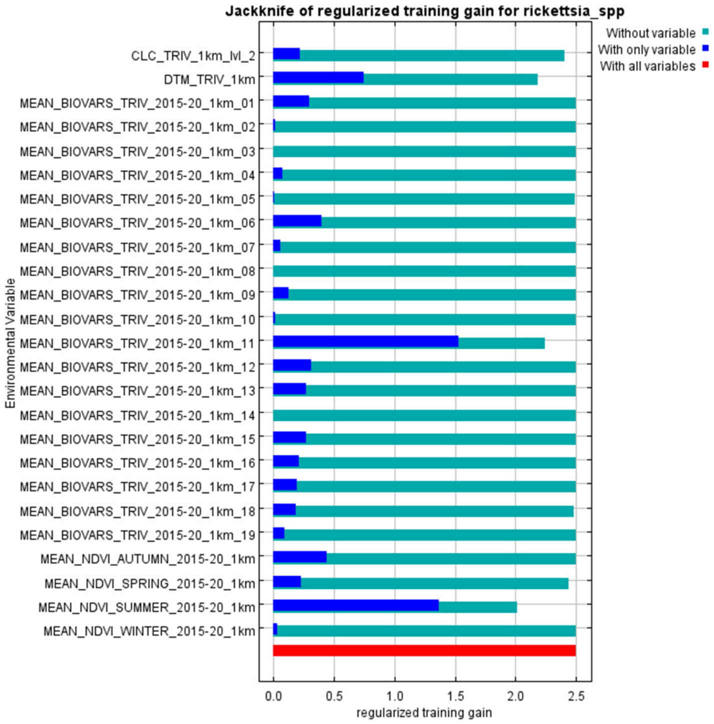

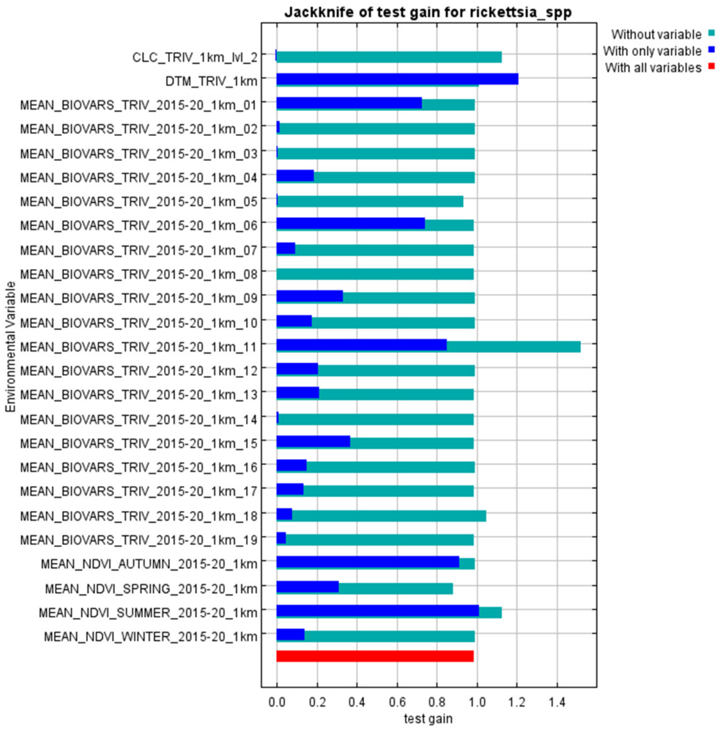
Jackknife of test gain for rickettsia_spp
Without variable
With only variable
With all variables
CLC_TRIV_1km_lvl_2
DTM_TRIV_1km
MEAN_BIOVARS_TRIV_2015-20_1km_01
MEAN_BIOVARS_TRIV_2015-20_1km_02
MEAN_BIOVARS_TRIV_2015-20_1km_03
MEAN_BIOVARS_TRIV_2015-20_1km_04
MEAN_BIOVARS_TRIV_2015-20_1km_05
MEAN_BIOVARS_TRIV_2015-20_1km_06
MEAN_BIOVARS_TRIV_2015-20_1km_07
MEAN_BIOVARS_TRIV_2015-20_1km_08
MEAN_BIOVARS_TRIV_2015-20_1km_09
MEAN_BIOVARS_TRIV_2015-20_1km_10
MEAN_BIOVARS_TRIV_2015-20_1km_11
MEAN_BIOVARS_TRIV_2015-20_1km_12
MEAN_BIOVARS_TRIV_2015-20_1km_13
MEAN_BIOVARS_TRIV_2015-20_1km_14
MEAN_BIOVARS_TRIV_2015-20_1km_15
MEAN_BIOVARS_TRIV_2015-20_1km_16
MEAN_BIOVARS_TRIV_2015-20_1km_17
MEAN_BIOVARS_TRIV_2015-20_1km_18
MEAN_BIOVARS_TRIV_2015-20_1km_19
MEAN_NDVI_AUTUMN_2015-20_1km
MEAN_NDVI_SPRING_2015-20_1km
MEAN_NDVI_SUMMER_2015-20_1km
MEAN_NDVI_WINTER_2015-20_1km
Environmental Variable
0.0 0.2 0.4 0.6 0.8 1.0 1.2 1.4
test gain

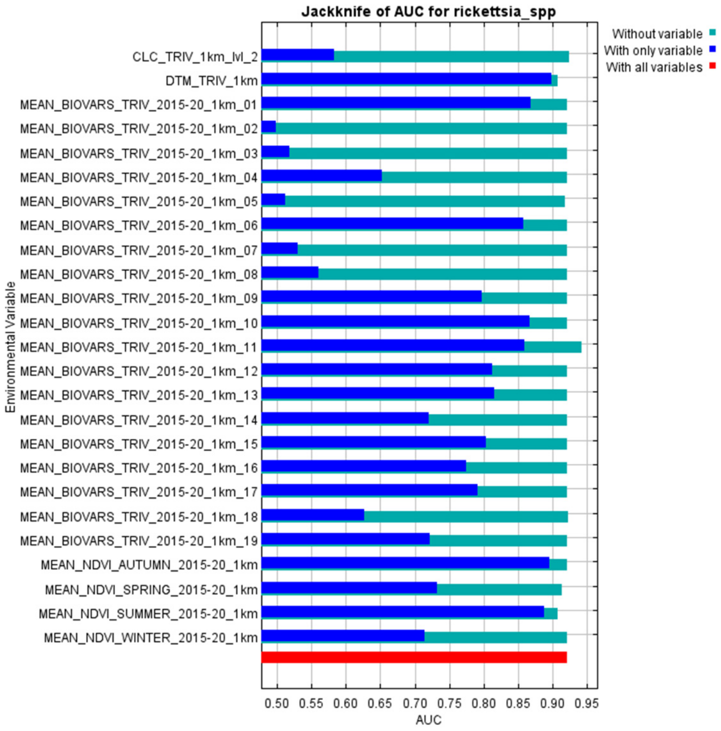
Jackknife of AUC for rickettsia_spp
Without variable
With only variable
With all variables
CLC_TRIV_1km_lvl_2
DTM_TRIV_1km
MEAN_BIOVARS_TRIV_2015-20_1km_01
MEAN_BIOVARS_TRIV_2015-20_1km_02
MEAN_BIOVARS_TRIV_2015-20_1km_03
MEAN_BIOVARS_TRIV_2015-20_1km_04
MEAN_BIOVARS_TRIV_2015-20_1km_05
MEAN_BIOVARS_TRIV_2015-20_1km_06
MEAN_BIOVARS_TRIV_2015-20_1km_07
MEAN_BIOVARS_TRIV_2015-20_1km_08
MEAN_BIOVARS_TRIV_2015-20_1km_09
MEAN_BIOVARS_TRIV_2015-20_1km_10
MEAN_BIOVARS_TRIV_2015-20_1km_11
MEAN_BIOVARS_TRIV_2015-20_1km_12
MEAN_BIOVARS_TRIV_2015-20_1km_13
MEAN_BIOVARS_TRIV_2015-20_1km_14
MEAN_BIOVARS_TRIV_2015-20_1km_15
MEAN_BIOVARS_TRIV_2015-20_1km_16
MEAN_BIOVARS_TRIV_2015-20_1km_17
MEAN_BIOVARS_TRIV_2015-20_1km_18
MEAN_BIOVARS_TRIV_2015-20_1km_19
MEAN_NDVI_AUTUMN_2015-20_1km
MEAN_NDVI_SPRING_2015-20_1km
MEAN_NDVI_SUMMER_2015-20_1km
MEAN_NDVI_WINTER_2015-20_1km
Environmental Variable
0.50 0.55 0.60 0.65 0.70 0.75 0.80 0.85 0.90 0.95
AUC

Results Maxent model for probability of presence of *Ehrlichia*-carrying ticks

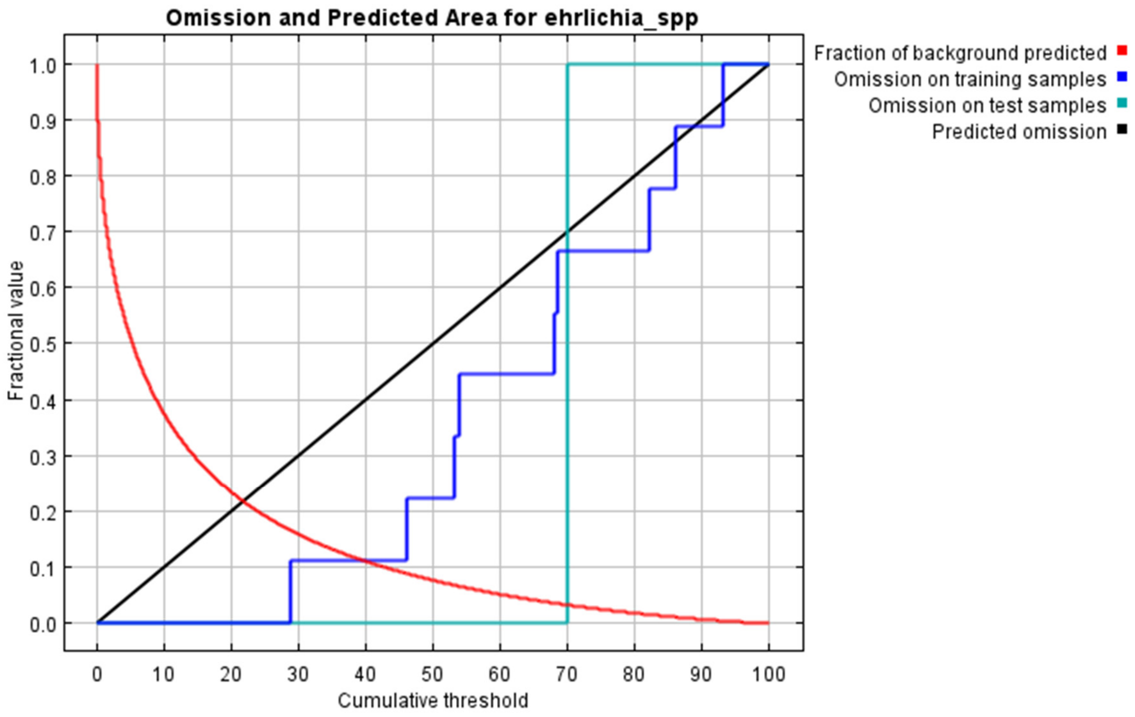

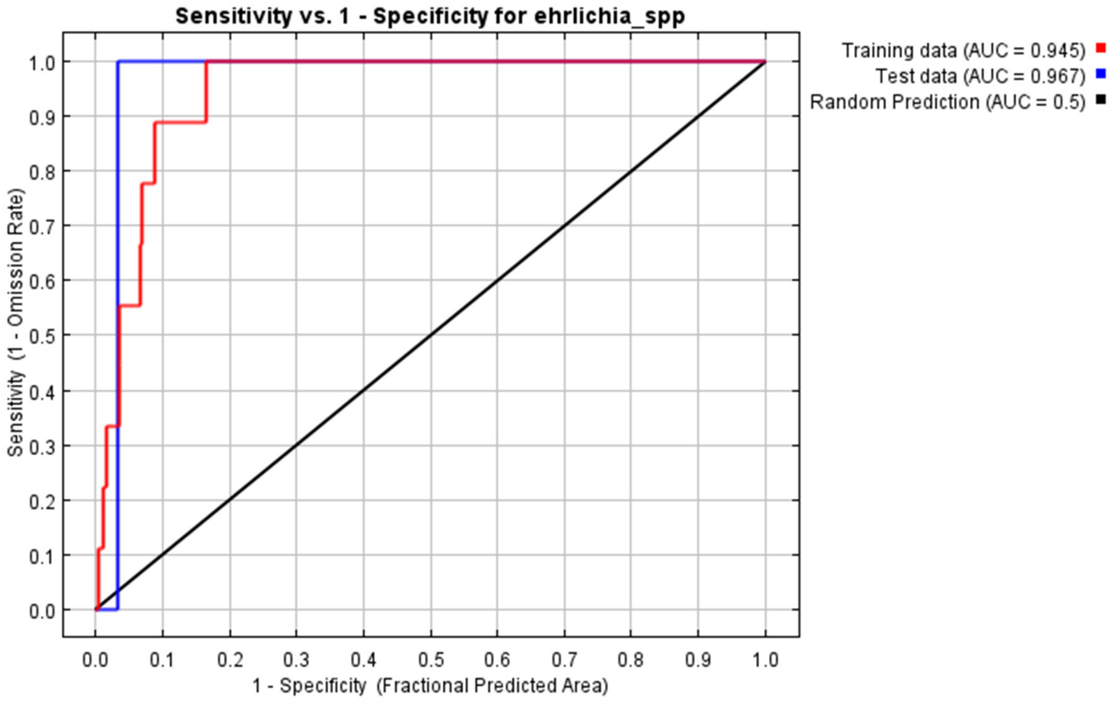

Variable	Percent contribution	Permutation importance
MEAN_NDVI_SUMMER_2015-20_1km	32	13.6
MEAN_BIOVARS_TRIV_2015-20_1km_13	14.7	0
MEAN_BIOVARS_TRIV_2015-20_1km_04	13.2	0
CLC_TRIV_1km_lvl_2	9.6	3.1
MEAN_BIOVARS_TRIV_2015-20_1km_07	9.2	42.9
DTM_TRIV_1km	7.2	7.9
MEAN_BIOVARS_TRIV_2015-20_1km_12	5.5	1.2
MEAN_BIOVARS_TRIV_2015-20_1km_15	4	0
MEAN_BIOVARS_TRIV_2015-20_1km_03	4	28.2
MEAN_BIOVARS_TRIV_2015-20_1km_18	0.4	3.1
MEAN_BIOVARS_TRIV_2015-20_1km_14	0	0
MEAN_BIOVARS_TRIV_2015-20_1km_05	0	0
MEAN_BIOVARS_TRIV_2015-20_1km_02	0	0
MEAN_BIOVARS_TRIV_2015-20_1km_01	0	0
MEAN_NDVI_WINTER_2015-20_1km	0	0
MEAN_NDVI_SPRING_2015-20_1km	0	0
MEAN_NDVI_AUTUMN_2015-20_1km	0	0
MEAN_BIOVARS_TRIV_2015-20_1km_19	0	0
MEAN_BIOVARS_TRIV_2015-20_1km_17	0	0
MEAN_BIOVARS_TRIV_2015-20_1km_16	0	0
MEAN_BIOVARS_TRIV_2015-20_1km_11	0	0
MEAN_BIOVARS_TRIV_2015-20_1km_10	0	0
MEAN_BIOVARS_TRIV_2015-20_1km_09	0	0
MEAN_BIOVARS_TRIV_2015-20_1km_08	0	0
MEAN_BIOVARS_TRIV_2015-20_1km_06	0	0

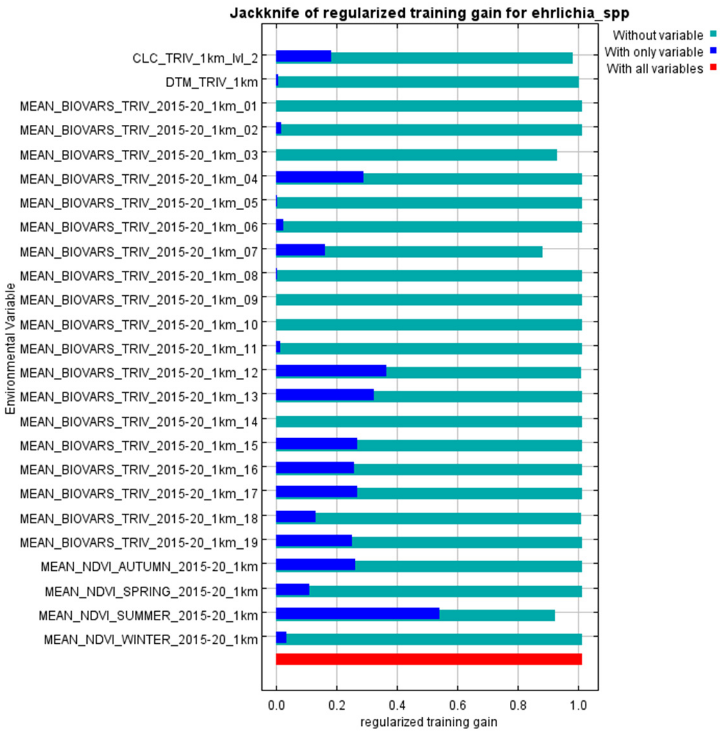

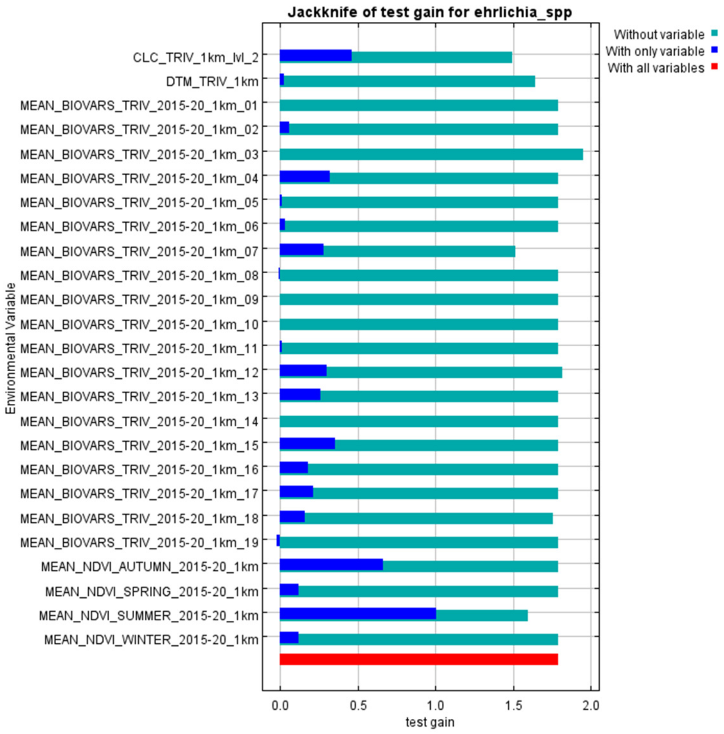
Jackknife of test gain for ehrlichia_spp
Without variable
With only variable
With all variables
Environmental Variable
CLC_TRIV_1km_lvl_2
DTM_TRIV_1km
MEAN_BIOVARS_TRIV_2015-20_1km_01
MEAN_BIOVARS_TRIV_2015-20_1km_02
MEAN_BIOVARS_TRIV_2015-20_1km_03
MEAN_BIOVARS_TRIV_2015-20_1km_04
MEAN_BIOVARS_TRIV_2015-20_1km_05
MEAN_BIOVARS_TRIV_2015-20_1km_06
MEAN_BIOVARS_TRIV_2015-20_1km_07
MEAN_BIOVARS_TRIV_2015-20_1km_08
MEAN_BIOVARS_TRIV_2015-20_1km_09
MEAN_BIOVARS_TRIV_2015-20_1km_10
MEAN_BIOVARS_TRIV_2015-20_1km_11
MEAN_BIOVARS_TRIV_2015-20_1km_12
MEAN_BIOVARS_TRIV_2015-20_1km_13
MEAN_BIOVARS_TRIV_2015-20_1km_14
MEAN_BIOVARS_TRIV_2015-20_1km_15
MEAN_BIOVARS_TRIV_2015-20_1km_16
MEAN_BIOVARS_TRIV_2015-20_1km_17
MEAN_BIOVARS_TRIV_2015-20_1km_18
MEAN_BIOVARS_TRIV_2015-20_1km_19
MEAN_NDVI_AUTUMN_2015-20_1km
MEAN_NDVI_SPRING_2015-20_1km
MEAN_NDVI_SUMMER_2015-20_1km
MEAN_NDVI_WINTER_2015-20_1km
0.0
0.5
1.0
1.5
2.0
test gain

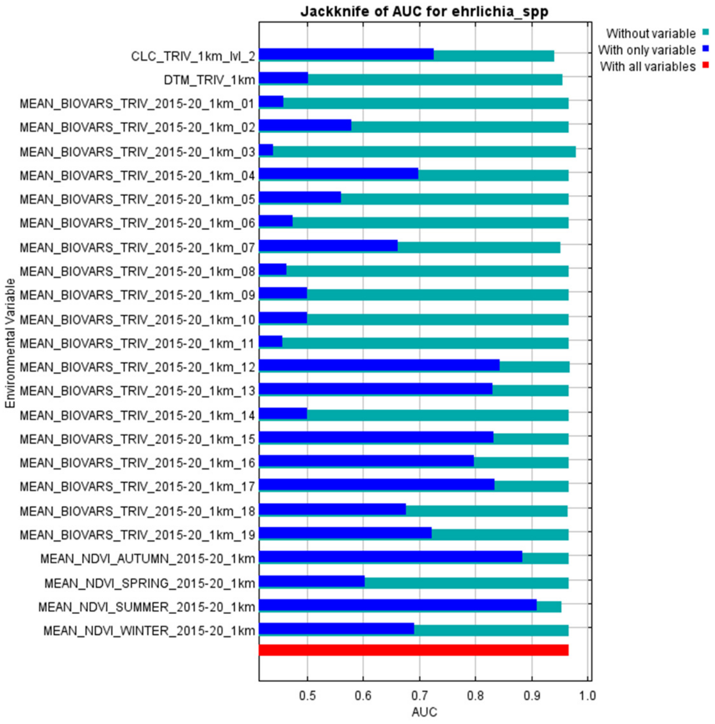
Jackknife of AUC for ehrlichia_spp
Without variable
With only variable
With all variables
Environmental Variable
CLC_TRIV_1km_lvl_2
DTM_TRIV_1km
MEAN_BIOVARS_TRIV_2015-20_1km_01
MEAN_BIOVARS_TRIV_2015-20_1km_02
MEAN_BIOVARS_TRIV_2015-20_1km_03
MEAN_BIOVARS_TRIV_2015-20_1km_04
MEAN_BIOVARS_TRIV_2015-20_1km_05
MEAN_BIOVARS_TRIV_2015-20_1km_06
MEAN_BIOVARS_TRIV_2015-20_1km_07
MEAN_BIOVARS_TRIV_2015-20_1km_08
MEAN_BIOVARS_TRIV_2015-20_1km_09
MEAN_BIOVARS_TRIV_2015-20_1km_10
MEAN_BIOVARS_TRIV_2015-20_1km_11
MEAN_BIOVARS_TRIV_2015-20_1km_12
MEAN_BIOVARS_TRIV_2015-20_1km_13
MEAN_BIOVARS_TRIV_2015-20_1km_14
MEAN_BIOVARS_TRIV_2015-20_1km_15
MEAN_BIOVARS_TRIV_2015-20_1km_16
MEAN_BIOVARS_TRIV_2015-20_1km_17
MEAN_BIOVARS_TRIV_2015-20_1km_18
MEAN_BIOVARS_TRIV_2015-20_1km_19
MEAN_NDVI_AUTUMN_2015-20_1km
MEAN_NDVI_SPRING_2015-20_1km
MEAN_NDVI_SUMMER_2015-20_1km
MEAN_NDVI_WINTER_2015-20_1km
0.5
0.6
0.7
0.8
0.9
1.0
AUC

SUPPLEMENTARY REFERENCES

[20] M. Schwaiger, P. Cassinotti, Development of a quantitative real-time RT-PCR assay with internal control for the laboratory detection of tick borne encephalitis virus (TBEV) RNA., J. Clin. Virol. Off. Publ. Pan Am. Soc. Clin. Virol. 27 (2003) 136–145. https://doi.org/10.1016/s1386-6532(02)00168-3.

[26] R Core Team. R: a language and environment for statistical computing. R Foundation for Statistical Computing, Vienna, Austria. https://www.R-project.org/. 2020 (accessed 13 February 2022).

[27] RStudio Team. RStudio: integrated development for R. RStudio, Inc., Boston, MA. http://www.rstudio.com/. 2019 (accessed 13 February 2022).

[43] Wood SN. Generalized Additive Models: An Introduction with R, 2nd edn. Boca Raton: Chapman and Hall/CRC (2017).

[44] Venables WN, Ripley B. D. Modern Applied Statistics with S, 4th edn. New York: Springer (2002).

[45] Wei T, Simko V. R package 'corrplot': Visualization of a Correlation Matrix (Version 0.92). https://github.com/taiyun/corrplot. 2021 (accessed 13 February 2022).

[46] Hijmans RJ. Raster: Geographic Data Analysis and Modeling. R package version 3.4-10. https://CRAN.R-project.org/package=raster. 2021 (accessed 13 February 2022).

[47] Wickham H. Ggplot2: elegant graphics for data analysis. 2nd edn. New York: Springer (2016).

[48] M. Gondard, S. Delannoy, V. Pinarello, R. Aprelon, E. Devillers, C. Galon, J. Pradel, M. Vayssier-Taussat, E. Albina, S. Moutailler, Upscaling the Surveillance of Tick-Borne Pathogens in the French Caribbean Islands, Pathogens 9 (2020). https://doi.org/10.3390/pathogens9030176.

[49] S.-M. Loh, A.W. Gofton, N. Lo, A. Gillett, U.M. Ryan, P.J. Irwin, C.L. Oskam, Novel Borrelia species detected in echidna ticks, Bothriocroton concolor, in Australia, Parasit. Vectors 9 (2016) 339. https://doi.org/10.1186/s13071-016-1627-x.

[50] L. Michelet, S. Delannoy, E. Devillers, G. Umhang, A. Aspan, M. Juremalm, J. Chirico, F.J. van der Wal, H. Sprong, T.P. Boye Pihl, K. Klitgaard, R. Bødker, P. Fach, S. Moutailler, High-throughput screening of tick-borne pathogens in Europe, Front. Cell. Infect. Microbiol. 4 (2014). https://doi.org/10.3389/fcimb.2014.00103.

[51] V.A. Rar, N. V Fomenko, A.K. Dobrotvorsky, N.N. Livanova, S.A. Rudakova, E.G. Fedorov, V.B. Astanin, O. V Morozova, Tickborne pathogen detection, Western Siberia, Russia., Emerg. Infect. Dis. 11 (2005) 1708–1715. https://doi.org/10.3201/eid1111.041195.

[52] R.L. Regnery, C.L. Spruill, B.D. Plikaytis, Genotypic identification of rickettsiae and estimation of intraspecies sequence divergence for portions of two rickettsial genes, J. Bacteriol. 173 (1991) 1576–1589. https://doi.org/10.1128/jb.173.5.1576-1589.1991.

[53] Y.-J. Choi, W.-J. Jang, J.-H. Kim, J.-S. Ryu, S.-H. Lee, K.-H. Park, H.-S. Paik, Y.-S. Koh, M.-S. Choi, I.-S. Kim, Spotted fever group and typhus group rickettsioses in humans, South Korea., Emerg. Infect. Dis. 11 (2005) 237–244. https://doi.org/10.3201/eid1102.040603.

[54] S. Centeno-Lima, V. Do Rosário, R. Parreira, A.J. Maia, A.M. Freudenthal, A.M. Nijhof, F. Jongejan, A fatal case of human babesiosis in Portugal: molecular and phylogenetic analysis, Trop. Med. Int. Heal. 8 (2003) 760–764. https://doi.org/https://doi.org/10.1046/j.1365-3156.2003.01074.x.

[55] V. Monteil, C. Salata, S. Appelberg, A. Mirazimi, Hazara virus and Crimean-Congo Hemorrhagic Fever Virus show a different pattern of entry in fully-polarized Caco-2

cell line., PLoS Negl. Trop. Dis. 14 (2020) e0008863.

https://doi.org/10.1371/journal.pntd.0008863.
